# Supplementary material for: Where there is no nurse: an observational study of large-scale mentoring of auxiliary nurses to improve quality of care during childbirth at primary health centres in India
Source: BMJ Open. 2019 Jul 9;9(7):e027147. doi: 10.1136/bmjopen-2018-027147 (PMC6615817; doi:10.1136/bmjopen-2018-027147)
Supplement: Author's manuscript [file bmjopen-2018-027147.draft_revisions.pdf]

BMJ Open is committed to open peer review. As part of this commitment we make the peer review history of every article we publish publicly available.

When an article is published we post the peer reviewers' comments and the authors' responses online. We also post the versions of the paper that were used during peer review. These are the versions that the peer review comments apply to.

The versions of the paper that follow are the versions that were submitted during the peer review process. They are not the versions of record or the final published versions. They should not be cited or distributed as the published version of this manuscript.

BMJ Open is an open access journal and the full, final, typeset and author-corrected version of record of the manuscript is available on our site with no access controls, subscription charges or pay-per-view fees (http://bmjopen.bmj.com).

If you have any questions on BMJ Open's open peer review process please email info.bmjopen@bmj.com

BMJ Open

Where there is no nurse – an observational study of large-scale mentoring of auxiliary nurses to improve quality of obstetric care at primary health centers in India.

Journal:	*BMJ Open*
Manuscript ID	bmjopen-2018-027147
Article Type:	Research
Date Submitted by the Author:	13-Oct-2018
Complete List of Authors:	Rao, Krishna; Johns Hopkins University Bloomberg School of Public Health, Department of International Health<br>Srivastava, Swati; Johns Hopkins University Bloomberg School of Public Health, International Health<br>Warren, Nicole; Johns Hopkins University School of Nursing<br>Mayra, Kaveri; Oxford Policy Management<br>Ahmed, Saifuddin; Johns Hopkins University Bloomberg School of Public Health, Population, Family And Reproductive Health
Keywords:	Quality in health care < HEALTH SERVICES ADMINISTRATION & MANAGEMENT, Health policy < HEALTH SERVICES ADMINISTRATION & MANAGEMENT, OBSTETRICS, PRIMARY CARE

SCHOLARONE™
Manuscripts

Title: Where there is no nurse – an observational study of large-scale mentoring of auxiliary nurses to improve quality of obstetric care at primary health centers in India.

Authors: Krishna D. Rao[1*], Swati Srivastava[1], Nicole Warren[2], Kaveri Mayra[3], Saifuddin Ahmed[4]

[1]Department of International Health, Johns Hopkins Bloomberg School of Public Health, Baltimore, Maryland, United States of America

[2]Johns Hopkins School of Nursing, Baltimore, Maryland, United States of America

[3]Oxford Policy Management, New Delhi, India

[4]Department of Population, Family and Reproductive Health, Johns Hopkins Bloomberg School of Public Health, Baltimore, Maryland, United States of America

[*]Corresponding author
Postal Address: Suite E-8148, Department of International Health, Johns Hopkins University, 315 N. Wolfe Street, Baltimore, MD 21215.
Email: kdrao@jhu.edu
Telephone Number: 443-432-9551

Word Count: 4,975 words

For peer review only

Abstract:

Objective: Scarcity of trained clinicians in LMICs often results in de-facto shifting of clinical tasks from higher to lower cadres which raises concerns about the quality of care. In human resource deficient regions of India, auxiliary nurse-midwifes (ANMs), who function as paramedical workers, manage deliveries at primary health centers, a function reserved for doctors or staff-nurses. This study examines if a long-term mentoring program improved the ability of ANMs to provide quality obstetric care.
Setting: Primary health centers in the state of Bihar, India; a total of 239 facilities surveyed and matched analysis based on 190 (134 intervention and 56 comparison) facilities.
Participants: Analysis based on 335 ANMs (237 mentored and 98 comparison) and 42 staff-nurses (28 mentored and 14 comparison).
Intervention: Large-scale mentoring program for a duration of six to nine months focused on ANMs and staff-nurses at primary health centers with the aim of improving the quality of basic emergency obstetric and newborn care.
Primary outcome measures: Nurse ability to provide correct actions in managing cases of normal delivery, postpartum hemorrhage, and neonatal resuscitation were assessed using a combination of clinical vignettes and Objective Structured Clinical Examinations.
Results: Mentoring improved the percentage of correct actions taken by ANMs to manage normal deliveries by 17.5 (95% CI: 14.8, 20.2), post-partum hemorrhage by 25.9 (95% CI: 22.4, 29.4), and neonatal resuscitation 28.4(95% CI: 23.2, 33.7) percentage points. There was no significant difference in the ability of mentored ANMs and staff-nurses. However, they provided only half the required correct actions. Ability was highest in the period immediately after mentoring and declined subsequently.
Discussion: Mentoring programs can improve the ability of ANMs to provide quality obstetric care and to levels that are comparable to trained nurses. However, mentoring has limited impact on sustaining quality practices. This makes continuing education programs critical.

Strengths and limitations of the study

- We were able to assess if the quality of obstetric care produced by auxiliary nurse-midwifes(ANM), who typically serve as paramedical workers in rural communities in India, can be improved through on-site mentoring to levels comparable with higher trained staff-nurses.
- A strength was that this was a large scale on-site mentoring program that was implemented in around 340 (60%) primary care facilities and included both ANMs and staff-nurses in the state of Bihar.
- Nurse ability measured using a unique tool that captures both knowledge and demonstrated skill.
- Observational study includes both intervention (mentored) and comparison (non-mentored) auxiliary nurse midwifes (ANM); reference to professional standards provided via observations of staff-nurse ability.
- Assumption that the comparison group ANMs and staff-nurses have the same ability level as those in the mentored group if they were not mentored.

For peer review only

For peer review only

INTRODUCTION

Access to a skilled birth attendant is a key strategy to reduce maternal deaths in low and middle-income countries. India's high maternal mortality of 167 per 100,000 live births contributes 19% of global maternal deaths[1,2]. Since 2005 institutional deliveries have been actively promoted by offering cash incentives to pregnant women (and health workers at public facilities), leading to the institutional delivery rate doubling between 2005 and 2014 (from 39% to 79%)[3]. Notably, nationwide around 52% (54% in rural) of the deliveries were in a public sector facility which highlights the important role of the public sector in providing obstetric care[3].

The remarkable increases in institutional deliveries have also raised concern about the quality of obstetric care. Studies have pointed out that despite substantial increases in institutional deliveries there has not been a concomitant reduction in maternal mortality[4–7]. While the infrastructure and resources available at health facilities have been an ongoing challenge, more recently, attention has also focused on the ability of health care providers[8–11]. A recent study of nurses' management of obstetric complications at public facilities in India found that only 14% of nurses conducted initial assessments, 58% made a correct clinical diagnosis, and 20% provided first-line care[11]. A variety of factors constrain the ability of nurses to provide quality care – inadequate infrastructure, poor training, limited access to continuing education and professional support from experienced clinicians, lack of agency in the workplace, and entrenched detrimental clinical practices[6,12–15].

In health system contexts where trained clinicians are scarce often there is a de-facto shifting of clinical responsibilities from higher to lower cadres which raises concerns about the quality of care [16, 17]. In the state of Bihar where this study is situated, the lack of doctors and staff-nurses at primary health centers (PHC) has resulted in lesser trained Auxiliary Nurse-midwives (ANM) providing obstetric care. Both staff-nurses and ANMs are carders within the public sector health system. Staff-nurses are required to have a diploma in General Nursing and Midwifery (GNM) which is a three year course in basic nursing. GNM nurses are trained to conduct normal deliveries and manage complications. ANMs are trained for two years. They were initially introduced into the health workforce in the 1950s to serve primarily as midwifes at PHCs and sub-centers[18]. However, overtime their function and training changed from that of a midwife to a paramedical health worker who provides a range of services such as family planning, immunizations and some level of antenatal care[18].

Efforts to improve skills of in-service nurses[1] have traditionally taken the form of short duration class-room style training sessions at a central location, which are not very effective for clinical skill building. On-site clinical mentoring programs, which involve longer term engagement and relationship building between mentor and mentee, have shown to be effective in improving nurse knowledge and skills around essential obstetric and neonatal care in other countries, and more recently in India[19–21]. When mentors become part of the mentee's environment, mentoring functions such as didactic instruction, demonstrations, and support and encouragement, have the potential to improve mentee skills and self-efficacy to perform effectively and overcome challenges. On-site mentoring programs for nurses at primary health care facilities have recently been attempted in several countries and in India; they report increased nurse knowledge and management of normal and complicated deliveries, increased adherence to quality protocols during delivery, and increased

[1] We use the term nurses to include both ANMs and staff-nurses.

For peer review only

confidence[22,23]. Further, mentoring was associated with a significant reduction in mortality for low birth weight babies[24].

This paper reports on a large-scale mentoring program to improve the ability of ANMs (and staff-nurses) to manage normal and complicated deliveries at PHCs in the state of Bihar, India. Specifically, this study investigates if mentoring improved the ability of ANMs to manage normal and complicated deliveries; how well mentored ANMs and staff-nurses compare in their ability to manage normal and complicated deliveries, and assess the sustainability of the mentoring effect. Nurse ability was assessed in managing standardized cases of normal delivery, postpartum hemorrhage, and neonatal resuscitation.

The state of Bihar (population 100 million) is among India's resource poor states. PHCs provide outpatient and occasionally inpatient care, maternal health services, family planning, public health services, and basic diagnostic and laboratory services. They cover between 20,000 to 30,000 people and are staffed with a doctor, nurses, and other supporting staff. PHCs are central to facility-based delivery of obstetric services because they are the first level in the public system which has clinicians trained to provide obstetric care such as doctors and nurses. In 2015-16, nearly 64% of all births were in health facilities and 36% at home; urban areas had 74% institutional births while rural areas had 63%[25]. Institutional births in public facilities have increased from just 4% of all institutional births in 2005-06 to almost 48% in 2015-16[25].

AMANAT nurse mentoring program in Bihar

CARE-India, a NGO, implemented the AMANAT on-site nurse mentoring program from May 2015 to January 2017 to improve the quality of basic emergency obstetric and newborn care (BEmONC) at primary health care facilities in the state of Bihar. Because the nomenclature of primary health care facilities is somewhat ambiguous in Bihar and change over time, we refer to AMANAT health facilities as PHCs in this study. The AMANAT program was preceded by a pilot from 2011 to 2013 in eight districts of Bihar[26]. The AMANAT mentoring program was implemented in four phases. In each phase 80 PHCs were purposively selected by CARE-India for mentoring. Several criteria determined selection of a PHC– availability of nurses or ANMs at the PHCs, the volume of deliveries, the infrastructural readiness of the PHC, the willingness of the PHC management to undertake mentoring, and the proximity of the PHC to other PHCs in the area such that mobile mentoring teams could easily rotate between them. In total 400 out of 534 block-level (the smallest administrative unit within a district) PHCs in Bihar received the nurse mentoring intervention.

In the AMANAT program a pair of nurse-mentors rotated multiple times through four PHCs over a six to nine-month period, spending one consecutive week at a PHC when they visited (with an average of four PHCs for each nurse-mentor pair). During their visit to the PHC, the nurse-mentors used structured learning sessions to provide didactic instruction covering a range of topics related to managing normal and complicated deliveries. Importantly, the nurse-mentors worked alongside mentees, observed them and provided instruction by demonstration through the co-management of cases. Nurses were trained on basic nursing procedures, infection prevention, basic obstetric and neonatal practices, management of complications such as post-partum hemorrhage, birth asphyxia, pre-eclampsia and others, documentation and reporting, team rapport and communication. Tailored, structured simulations were used for normal deliveries and complications management through the

For peer review only - http://bmjopen.bmj.com/site/about/guidelines.xhtml

PRONTOPack and other simulation kits[27]. These kits include MamaNatalie, a birth simulator worn by a demonstrator to resemble a pregnant woman and used for obstetric practices, and NeoNatalie, a neonate model used to demonstrate neonatal practices.

METHODS

Study design

The AMANAT program was implemented in four phases, such that, in each phase 80 PHCs were purposively selected. Of the approximately 534 PHCs in Bihar, 80 PHCs were exposed to mentoring in a pilot phase (2011- 2013) and a further 320 were subsequently exposed during the AMANAT program (2015-2016). Therefore, around 134 PHCs were not exposed to mentoring. This study focuses on the 240 PHCs in phases 2, 3 and 4. These PHCs were first observed just after mentoring was completed and followed-up twice after that. We excluded phase-1 PHCs because they had completed mentoring nearly two years prior to the survey date and we expected significant number of transfers of mentored nurses during this time. To assess the effect of mentoring on nurse ability we use a quasi-experimental post-test with matched comparison group. PHCs exposed to AMANAT mentoring were matched on several criteria with non-mentored PHCs.

Selection of PHCs: The 240 PHCs in phase 2,3 and 4 of the AMANAT program served as our sampling frame. For Phases 2 and 3, which began mentoring at approximately the same time, 40 PHCs from each phase were randomly sampled and a total of 79 out of these 80 selected PHCs were surveyed.[2] From Phase 4, 80 PHCS were selected and all were surveyed. Thus, a total of 159 mentored PHCs constitute the pool of intervention group PHCs

134 PHCs in Bihar did not receive any mentoring either through the pilot or AMANAT program. 7 of these PHCs reported 15 or less deliveries per month in the state Health Management Information System (HMIS) and were excluded. The remaining 127 PHCs were mapped to assess geographical proximity to mentored facilities. From these PHCs, 80 PHCs that were closest to a mentored PHC – either from the same block or adjacent block as an intervention PHC - were purposively selected. These 80 PHCs form the pool of comparison group PHCs.

Selection of nurses: For each sampled PHC the mentored staff-nurses or ANMs were identified from mentoring rosters. From this list two were randomly selected and were requested to be present on the day the survey team arrived. From the 159 mentored PHCs a total of 314 staff-nurses and ANMs were interviewed (out of a target of 318) and from the 80 comparison PHCs 160 staff-nurses and ANMs were interviewed (out of a target of 160).

Data collection: Data for the study was collected between September 2016 and November 2017. Further, in 2016, as part of a larger study, a facility assessment was carried out on the service delivery readiness of PHCs in Bihar. All PHCs (and other health facilities) were surveyed and information on availability and condition of drugs, supplies, equipment, and building condition. Data was collected using tablets. The statistical analysis was conducted using Stata 14[28].

---

[2] One facility in Phase 2 and 3 received the intervention in the initial pilot phase between Jan 2011 and December 2013 and was excluded.

Ethical approval: Ethical clearance for the study was received from the Centre for Media Studies (New Delhi) Institutional Review Board vide approval number IRB00006230.

Patient and public involvement: Nurses were consulted in developing study protocols and training data collectors. The data collectors were also trained nurses. Feedback from nurses during pre-testing enabled designing study protocols so that they do not to place undue time burden on respondent nurses and to be flexible to demands of their professional work.

Analytical methods

Matching health facilities: The health facility survey collected information on the service delivery readiness of PHCs in Bihar. Using the service delivery/readiness domains indicated in the WHO's Service Availability and Readiness Assessment (SARA)[29] as a guide, we identified the following six indicators for matching PHCs: location – connected to metaled road; basic amenities – regular power supply available or made available through a generator, hand washing station with running water available in labor room; drugs – oxytocin present; infrastructure – sanctioned bed strength; service utilization – average number of deliveries per year in 2016 (from HMIS).

We use Coarsened Exact Matching (CEM) to match PHCs in intervention and comparison groups[30]. After matching, 134 (84%) of the 159 AMANAT PHCs were matched with 56 (70%) of the 80 PHCs in the non-mentored group. From these matched PHCs, there were 265 nurses in the intervention group and 112 in the comparison group.

Measuring nurse ability: To measure nurse ability, we developed a tool that captures both their knowledge and demonstrated skill in managing normal and complicated deliveries. Our tool adapts elements of clinical vignettes with Objective Structured Clinical Examinations (OSCEs). Clinical vignettes have been used to measure health worker knowledge in treating specific disease conditions in the context of a simulated consultation[31–33]. Health care providers are presented with standardized cases specific to a disease condition and their knowledge in managing the specific condition is evaluated using checklists. OSCEs are commonly used across nursing and other allied health professions and involve learners rotating through structured, time-limited stations to demonstrate discrete skills[34]. OSCEs are used extensively in many countries to assess competencies in obstetric and newborn care, including in India[35–39]. Our tool combined clinical vignettes and OSCEs by evaluating knowledge (via verbal responses) and skills (via demonstration) in managing normal and complicated deliveries. Nurses and ANMs were evaluated in how they would manage three standardized cases - normal birth, neonatal resuscitation, and postpartum hemorrhage. These cases were selected because they were taught to all mentees and are important to maternal and newborn health outcomes in India. Tool development was facilitated by CARE program developers, India-based obstetric experts, and international experts in BEmONC training.

The three cases on which nurses and ANMs were evaluated – normal delivery, post-partum hemorrhage, and neonatal resuscitation – were structured to assess ability in history taking, examinations, case identification/diagnosis, and management. Two interviewers administered each case – interviewer #1 played the role of the pregnant woman and wore a Mama Natalie, and interviewer #2 observed the interaction using a standard checklist. Each case began with the respondent being presented with a situation by interviewer #2 – for example, the normal delivery case is introduced to the nurse/ANM as "*This is Tara [pointing to interviewer #1]. She is pregnant. She has come to see you at the clinic.*" The respondent is then asked about what history questions she will ask

For peer review only

Tara, what examinations will be done, and the respondent will demonstrate them on the Mama Natalie. To each relevant history question or examination done, the respondent receives a standard response. For instance, if the respondent asks "When did you have your last menstrual period (LMP)?" a standard response of "nine months" is offered or if the respondent demonstrates checking for fetal heart sound a response of "30" is given. No responses outside of the pre-structured conversation are given. Based on the results of the history and examinations, the nurse is then asked what the diagnosis/clinical decision is (e.g. normal or complicated delivery), and then to demonstrate on the Mama Natalie how she would manage the delivery. Each case comprises a set of items that correspond to correct actions in the domains of history & examinations, case detection/diagnosis, and case management. All relevant correct actions were marked off on a checklist.

Data analysis: Each case comprises a set of items that correspond to correct actions necessary for managing the case. The unit of analysis is a necessary action (verbal or demonstration) provided by the respondent in the course of completing a case. Exploratory data analysis (e.g. histograms) was conducted to examine the distribution of provider responses across PHCs and nurses. Facilities with extreme values were identified and scrutinized. We then computed the proportion of relevant correct actions in history, examination, case detection/diagnosis, and management sections for each of the three cases. Next, using logistic regressions, for each case, we modelled the probability of a nurse providing a correct action as a function of mentoring status, nurse characteristics (GNM nurse (ref: ANM), age, permanent employee (ref: contractual), facility characteristics (connected to metaled road, regular power supply available or made available through a generator, hand washing station with running water available in labor room, oxytocin present; sanctioned bed strength, average number of deliveries per year in 2016), and item dummy variables. Regression standard errors were adjusted for clustering. Goodness of fit test was performed on the final model by inspecting plots of predicted versus observed values. Residual plots were examined to identify outliers and influential observations. Regression results are presented in terms of marginal effects.

RESULTS

Table 1 shows the characteristics of the mentored and comparison PHCs and nurses in the matched and full sample. The full sample (see Total sample) includes the 238 mentored and comparison PHCs before matching was carried out. The matched mentored and comparison PHCs are broadly similar in their characteristics indicating that matching was successful. For the average number of deliveries in the past year, however, mentored PHCs receive more visits on average than the comparison ones. The PHCs and nurses in the full sample of facilities (i.e. Total sample in Table 1), are also broadly similar to the PHCs included in the matched sample in terms of PHC and nurse characteristics, though the matched PHCs have slightly higher levels on all the PHC level indicators.

[Table 1 here]

There are several notable points about the characteristics of nurses. First, the overwhelming majority of nurses were ANMs and staff-nurses are a minority. The mentored group had a higher proportion of ANMs relative to the comparison group. Secondly, the majority of nurses were regular staff (as opposed to contractual staff); their proportion is higher in the comparison group. Finally, nurses

For peer review only

across groups are similar in average age. Nurse age is also correlated with service duration; nurses in the mentored and comparison groups had similar practice durations.

Nurses in the AMANAT program typically experienced six months of mentoring. Overall 93% of nurses reported having at least six months of mentoring exposure (Table 1). Duration of mentoring ranged from two to nine months, with an average of seven months of mentoring. These results suggest that there was high adherence to the AMANAT program among those who participated.

ANM and Staff-nurse ability

In all three cases mentored ANMs and staff-nurses provided significantly more correct actions than the comparison group (Table 2). For example, mentored (comparison) ANMs provided 53%(36%) correct actions for the normal delivery case, a percentage point difference of 17 points; 49%(24%) for the post-partum hemorrhage case, a percentage point difference of 29 points, and 53%(25%) for the neonatal resuscitation case, a percentage point difference of 28 points. However, the overall proportion of correct actions among mentored ANMs and staff nurses does not exceed 60% in any case. Table 2 also shows the predicted probabilities from the regressions with respondent and PHC characteristics at their reference values. For example, for the normal delivery case mentored (comparison) ANMs provided 53%(36%) of the correct actions, while mentored staff nurses (comparison) provided 55% (42%) correct actions. Notably, the 95% confidence intervals between mentored and comparison nurses do not overlap - mentored ANMs and staff nurses had significantly higher percentage of correct actions compared to the comparison group.

[Table 2 here]

These overall trends are mirrored in staff-nurse and ANM performance across the ability domains (Table 3). Mentored ANMs and staff nurses demonstrated significantly more correct actions for history & examinations, and case management relative to those in the comparison group. Mentored (comparison) ANMs provided 54% (41%)of the correct history and examination actions, 63% (34%) for post-partum hemorrhage and 55%(28%) for neonatal resuscitation. Similarly, mentored nurses performed better than the comparison group – mentored (comparison) ANMs provided 51% (28%) of correct case management actions for normal delivery, 37%(16%) for post-partum hemorrhage, and 51%(22%) for neonatal resuscitation. These patterns are also seen in the performance of staff nurses.

[Table 3 here]

The majority of ANMs and staff-nurses were able to correctly identify cases (except for ANMs in the comparison group for neonatal resuscitation case) (Table 3). Expectedly, the percent of correct responses was highest for the normal delivery case since the pre-service training of both ANMs and staff nurses include the basics of managing normal delivery. However, the ability to correctly identify a case was generally higher among mentored nurses (Table 3). Mentored (comparison) ANMs correctly identified 96%(99%) of the normal delivery cases, 84%(51%) of the post-partum hemorrhage case, and 83%(50%) of the neonatal resuscitation case. Similarly, mentored staff-nurses had higher case identification than the comparison group except for the normal delivery case. In general, staff-nurses did much better than ANMs in correctly identifying a particular case.

For peer review only

Of interest is to see how well ANMs compare with staff nurses in their ability to manage normal and complicated deliveries. Comparison group (i.e. those without any mentoring) ANMs had significantly lower ability relative to staff-nurses for all three cases. However, ANMs that experienced mentoring have similar levels of performance as staff nurses on all three cases. For example, for the normal delivery case mentored ANMs (staff nurses) provided 53%(55%) correct actions, 50%(50%) for post-partum hemorrhage, and 53%(57%) for neonatal resuscitation (Table 2). The overlapping confidence intervals between mentored ANMs and staff nurses indicates that there are no statistically significant differences between them.

Effect of mentoring

Mentored ANMs and staff nurses have significantly greater proportion, relative to the comparison group, of correct actions overall, and for the domains of history & examinations, case identification (except for normal delivery), and in case management (Table 2, 3). Figure 1 illustrates the adjusted mentoring effect (i.e. treatment effect on the treated) on ANMs and staff-nurses. These are marginal effects from the regression models with other variables at their reference values. ANMs in the mentored group had 17.5% more correct actions for normal delivery relative to the comparison group, 25.9% more for post-partum hemorrhage, and 28.4% more for neonatal resuscitation. Staff nurses in the mentored group also show similar levels of improved performance. Notably, in all cases the mentoring effect is statistically significant i.e. the 95% confidence intervals do not overlap zero.

[Figure 1 here]

Both ANMs and staff nurses appear to have gained from mentoring, but ANMs have gained more. As seen in Figure 1, the mentoring effect for each case is greater for ANMs than staff-nurses. Finally, because no differences in the proportion of correct actions are seen between mentored ANMs and staff nurses (Table 2), it appears that mentoring brought ANMs on par with staff nurses in the management of normal deliveries, post-partum hemorrhage, and neonatal resuscitation.

Of interest is to know if the mentoring effect on nurse ability maintains itself or declines with time. Figure 2 illustrates this by plotting estimates from local area regressions and shows the probability of nurses demonstrating a correct action at different points in time after mentoring was completed. Nurses from the same group of PHCs were followed over time. In all three cases, nurse ability declines with time. In the normal delivery case, just after mentoring a nurse was able to provide 53% of the correct actions, which declined to 43%, a ten-percentage point drop, 16 months after mentoring was completed. Similar trends are seen in the case of neonatal resuscitation and postpartum hemorrhage. In the neonatal resuscitation case, the slight increase seen at the tail end appears to be an artifact of the data. For this case, nurses just after mentoring were able to provide 55% of the correct actions, which declined to 44% after 16 months post mentoring. For post-partum hemorrhage, just after mentoring nurses were able to provide 50% of correct actions, which fell to 37% post mentoring, a drop of 13 percentage points.

[Figure 2 here]

DISCUSSION

For peer review only

In India's public sector health system ANMs function as community based paramedical health workers and provide a range of services such as family planning, immunizations and antenatal care[18]. In human resource scare states like Bihar, where there is a scarcity of trained doctors and staff-nurses, ANMs are the de-facto providers of obstetric care at many primary care facilities even though they are not formal trained for this role. In our study which covered 60% of the 534 PHCs (or similar facilities) in Bihar, staff-nurses were present in only 13% of the PHCs. In a policy environment that aggressively promotes institutional deliveries the quality of care provided by ANMs is an important concern.

A principal finding from this study is that mentoring improved the ability of ANMs (and staff-nurses) at PHCs to manage normal and complicated deliveries. In all three cases – normal delivery, postpartum hemorrhage, and neonatal resuscitation – mentored ANMs and staff-nurses had better ability compared to non-mentored ones. This finding is consistent with evaluations of mentoring programs in other contexts and India[20,21]. Mentored ANMs (staff nurses) were able to provide 17% (13%) more correct actions for managing normal deliveries, 26%(14%) more for postpartum hemorrhage, and 28%(19%) more correct actions for neonatal resuscitation, compared to their non-mentored colleagues. The gains were greater for ANMs since they started from a lower level of ability relative to staff nurses. Among these three cases, for both ANMs and staff nurses the largest gain in ability was in neonatal resuscitation, followed by postpartum hemorrhage, and normal delivery. This is along expected lines since both ANMs and staff nurses have exposure to management of normal deliveries during pre and in-service training and so the incremental effect of mentoring would be relatively smaller. Further, the AMANAT mentoring program emphasized management of complications such as postpartum hemorrhage and neonatal resuscitation, which explains the gains observed in this area.

ANMs that had completed mentoring had the same ability as staff nurses to manage normal and complicated deliveries. Further, un-mentored ANMs had significantly poorer ability than un-mentored staff-nurses. This mentored ANM achievement is particularly salient because ANMs typically do not provide obstetric care and operate as paramedical health workers providing services such as family planning, immunizations and some level of antenatal care[18]. It is unlikely that improvements in ANM ability would be achievable outside a mentoring context. The AMANAT program provided a combination of didactic teaching, supportive supervision, and professional support over a long period of time in the context of a mentor-mentee relationship. This is more potent in improving ability and self-efficacy than the typical class room style trainings that health workers are usually exposed to.

Mentoring improved case identification among ANMs. The vast majority of mentored ANMs were able to correctly identify a normal delivery case, 84% correctly identified a postpartum hemorrhage case, and 83% the neonatal resuscitation case. While non-mentored ANMs and staff-nurses performed similarly as mentored ANMs in correctly identifying normal deliveries, they did quite poorly in identifying the other two cases. For example, among non-mentored ANMs, only half of them were able to correctly identify the postpartum hemorrhage or neonatal resuscitation case. The biggest improvement in correctly identifying a case was among ANMs for postpartum hemorrhage and neonatal resuscitation.

It is important to be cautious about the extent to which quality of care can be improved by one-off mentoring programs. Even after six to nine months of mentoring through the AMANAT program

For peer review only

there remained considerable scope for improving quality of care. Mentored ANMs and staff-nurses were able to provide a little more than half the correct actions on all three cases. This is also reflected in the ability domains of history & examinations and case management on which nurses were evaluated. For example, mentored ANMs (nurses) were able to provide only 51% (54%) of correct actions for case management in the normal delivery case, 37%(38%) in the postpartum hemorrhage case, and 51%(56%) correct actions in the neonatal resuscitation case.

Another reason for being cautious is that the mentoring effect on nurse ability appears to decay over time. In all three cases we find that nurse ability was highest in the period immediately after mentoring and this declined with the passage of time. The decay was least for the normal delivery case and most for the neonatal resuscitation case. This has also been observed in other nurse mentoring studies in India, with a greater decline in desirable neonatal and obstetric practices and a slower decline in nurse knowledge[22,40]. These patterns highlight the importance of continuing health worker education and make a strong case for mentoring or similar programs to be offered periodically to health workers.

A limitation of this study is that we do not observe the ability of nurses before they started mentoring. As such, our estimates of the mentoring effect will be biased if the matched comparison group nurses do not have the same average ability as those in the mentored group before they were mentored. There are several reasons why this may not be the case. One source of bias could arise from differences in nurse characteristics between mentored and comparison groups that are related to their ability. Both mentored and comparison group ANMs (or staff-nurses) had the same standard pre-service training and require the same qualifications to be recruited into government service. As such, it is unlikely that the extent or quality of nurse pre-service training differed between groups. Further, in our regression we control for difference in nurse characteristics (in addition to facility characteristics) between groups. As such estimates of the mentoring effect are adjusted for differences in nurse characteristics such as age, years of service, and employee type between mentored and non-mentored nurses.

Nurse ability can vary by the characteristics of their work location and environment. For example, studies have noted that the ability of clinical care providers varies with the economic status of the community i.e. better-off areas have better quality providers[41]. Similarly, nurses working in a facility with good infrastructure might have better ability. As such, differences in the facility location or characteristics between the mentored and comparison groups can bias estimates of the mentoring effect on nurse ability. We created the comparison group PHCs to be as similar as possible to the mentored PHCs in terms of location and physical environment. Comparison group PHCs were selected such that they were in close geographic proximity (from the same of adjacent administrative block) to the mentored PHCs. Further, we matched mentored and comparison PHCs on location, service readiness, and PHC use criteria.

Just because nurses have been mentored it does not automatically follow that they also have improved practice in managing deliveries. However, studies that have examined the association between ability and practice report that these measures are correlated, though there is a typically a gap between what clinicians know and what they do (i.e. practice)[42,43]. In general, more able clinicians also "do more" in practice, and ability measures the upper bound of what is possible in practice.

For peer review only

Mentoring programs can improve the quality of obstetric care of lesser trained health workers like ANMs to levels comparable to those of staff-nurses who have higher levels of training. In health systems which struggle to recruit highly trained health workers to underserved areas, such mentoring programs offer a way of improving the quality of care. However, the effect of such mentoring is not permanent and decays with time. This makes it important to expose health workers to regular continuing education programs.

For peer review only

ACKNOWLEDGEMENTS

The authors would like to acknowledge Priya Nanda (BMGF, Delhi), Yamini Atmavilas (BMGF Delhi), Dr. Sridhar Srikantiah (CARE-Bihar), Dr. Tanmay Mahapatra (CARE-Bihar), Dr. Aboli Gore (CARE-Bihar), Tom Newton-Lewis (Oxford Policy Management, Delhi).

COMPETING INTERESTS

The authors declare they have no competing interests.

FUNDING

The study was funded by the Bill and Melinda Gates Foundation (https://www.gatesfoundation.org/) via grant number OPP1142884. The funders had no role in study design, data collection and analysis, decision to publish, or preparation of the manuscript.

AUTHORS CONTRIBUTIONS

The study was conceived, and designed by KDR, NW, and SA. Study protocols were developed and by KDR, NW, SS, and KM. Field work was conducted by KDR, NW, SS, and KM. Data analysis was done by KDR, SS and SA. The paper was written primarily by KDR, SS and NW, with contributions from KM and SA.

DATA SHARING

The data contained within this study can be obtained by writing to kdrao@jhu.edu.

REFERENCES

1. Sample Registration Survey 2011-13 Census of India. Maternal Mortality Ratio(MMR), Maternal Mortality Rate and Life Time Risk; India, EAG & Assam, South and Other states, 2011-13 [Internet]. [cited 2018 Apr 28]. Available from: http://www.censusindia.gov.in/vital_statistics/mmr_bulletin_2011-13.pdf
2. World Health Organization. Trends in maternal mortality: 1990 to 2015: estimates by WHO, UNICEF, UNFPA, World Bank Group and the United Nations Population Division [Internet]. Geneva; 2015 [cited 2018 Apr 28]. Available from: www.who.int/about/licensing/
3. International Institute for Population Sciences. National Family Health Survey-4 2015-16 – India fact sheet [Internet]. Mumbai; [cited 2018 Apr 28]. Available from: http://rchiips.org/nfhs/pdf/NFHS4/India.pdf
4. Randive B, Diwan V, De Costa A. India's Conditional Cash Transfer Programme (the JSY) to Promote Institutional Birth: Is There an Association between Institutional Birth Proportion and Maternal Mortality? PLoS One. 2013;8(6).
5. Sabde Y, Diwan V, Randive B, Chaturvedi S, Sidney K, Salazar M, et al. The availability of emergency obstetric care in the context of the JSY cash transfer programme in Madhya Pradesh, India. BMC Pregnancy Childbirth [Internet]. 2016 [cited 2018 Apr 30];16. Available from: https://bmcpregnancychildbirth.biomedcentral.com/track/pdf/10.1186/s12884-016-0896-x
6. Chaturvedi S, Randive B, Diwan V, De Costa A, Baradaran HR. Quality of Obstetric Referral Services in India's JSY Cash Transfer Programme for Institutional Births: A Study from Madhya Pradesh Province. PLoS One [Internet]. 2014 [cited 2018 Apr 25];9(5). Available from: http://journals.plos.org/plosone/article/file?id=10.1371/journal.pone.0096773&type=printable
7. Ameh C, Msuya S, Hofman J, Raven J, Mathai M, Van Den Broek N. Status of Emergency Obstetric Care in Six Developing Countries Five Years before the MDG Targets for Maternal and Newborn Health. [cited 2018 Apr 24]; Available from: http://journals.plos.org/plosone/article/file?id=10.1371/journal.pone.0049938&type=printable
8. Mony PK, Krishnamurthy J, Thomas A, Sankar K, Ramesh BM, Moses S, et al. Availability and Distribution of Emergency Obstetric Care Services in Karnataka State, South India: Access and Equity Considerations. PLoS One [Internet]. 2013 [cited 2018 Apr 25];8(5). Available from: http://journals.plos.org/plosone/article/file?id=10.1371/journal.pone.0064126&type=printable
9. Sharma G, Powell-Jackson T, Haldar K, Bradley J, Filippi V. Quality of routine essential care during childbirth: clinical observations of uncomplicated births in Uttar Pradesh, India. Bull World Health Organ [Internet]. 2017;95(6):419–29. Available from: http://www.who.int/entity/bulletin/volumes/95/6/16-179291.pdf
10. Jayanna K, Mony P, Bm R, Thomas A, Gaikwad A, Hl M, et al. Assessment of facility readiness and provider preparedness for dealing with postpartum haemorrhage and pre-eclampsia/eclampsia in public and private health facilities of northern Karnataka, India: a cross-sectional study. [cited 2018 Apr 2]; Available from: http://www.biomedcentral.com/1471-2393/14/304
11. Chaturvedi S, Upadhyay S, De Costa A. Competence of birth attendants at providing

emergency obstetric care under India's JSY conditional cash transfer program for institutional delivery: an assessment using case vignettes in Madhya Pradesh province. [cited 2018 Apr 25]; Available from: http://www.biomedcentral.com/1471-2393/14/174
12. Evans CL, Maine D, McCloskey L, Feeley FG, Sanghvi H. Where there is no obstetrician - increasing capacity for emergency obstetric care in rural India: An evaluation of a pilot program to train general doctors. Int J Gynecol Obstet [Internet]. 2009 Dec [cited 2018 Feb 19];107(3):277–82. Available from: http://www.ncbi.nlm.nih.gov/pubmed/19846091
13. Iyengar SD, Iyengar K, Martines JC, Dashora K, Deora KK. Childbirth practices in rural Rajasthan, India: Implications for neonatal health and survival. J Perinatol. 2008;28(SUPPL. 2):23–30.
14. Iyengar K, Jain M, Thomas S, Dashora K, Liu W, Saini P, et al. Adherence to evidence based care practices for childbirth before and after a quality improvement intervention in health facilities of Rajasthan, India. BMC Pregnancy Childbirth. 2014;14(1):1–12.
15. Iyengar K, Iyengar SD. Emergency obstetric care and referral: experience of two midwife-led health centres in rural Rajasthan, India. Reprod Health Matters [Internet]. 2009 May 1 [cited 2018 Apr 25];17(33):9–20. Available from: https://www.sciencedirect.com/science/article/pii/S096880800933459X
16. Rao KD, Shahrawat R, Bhatnagar A. Composition and distribution of the health workforce in India: estimates based on data from the National Sample Survey. WHO South-East Asia J public Heal. 2016 Sep;5(2):133–40.
17. Rao KD, Sundararaman T, Bhatnagar A, Gupta G, Kokho P, Jain K. Which doctor for primary health care? Quality of care and non-physician clinicians in India. Soc Sci Med [Internet]. 2013 May 1 [cited 2018 Apr 30];84:30–4. Available from: https://www.sciencedirect.com/science/article/pii/S0277953613000919?via%3Dihub
18. Mavalankar D, Vora KS. The Changing Role of Auxiliary Nurse Midwife (ANM) in India : Implications for Maternal and Child Health (MCH) Dileep Mavalankar The Changing Role of Auxiliary Nurse Midwife (ANM) in India : Implications for Maternal and Child Health (MCH). 2008;(SEPTEMBER 2015).
19. Anatole M, Magge H, Redditt V, Karamaga A, Niyonzima S, Drobac P, et al. Nurse mentorship to improve the quality of health care delivery in rural Rwanda. Nurs Outlook [Internet]. 2013 May 1;61(3):137–44. Available from: http://dx.doi.org/10.1016/j.outlook.2012.10.003
20. Fischer EA, Jayana K, Cunningham T, Washington M, Mony P, Bradley J, et al. Nurse Mentors to Advance Quality Improvement in Primary Health Centers: Lessons From a Pilot Program in Northern Karnataka, India. [cited 2018 Apr 2]; Available from: http://www.ghspjournal.org/content/ghsp/3/4/660.full.pdf
21. Jayanna K, Bradley J, Mony P, Cunningham T, Washington M, Bhat S, et al. Effectiveness of Onsite Nurse Mentoring in Improving Quality of Institutional Births in the Primary Health Centres of High Priority Districts of Karnataka, South India: A Cluster Randomized Trial. [cited 2018 Apr 2]; Available from: http://journals.plos.org/plosone/article/file?id=10.1371/journal.pone.0161957&type=printable
22. Das A, Nawal D, Singh MK, Karthick M, Pahwa P, Shah MB, et al. Evaluation of the mobile nurse training (MNT) intervention - a step towards improvement in intrapartum practices in Bihar, India. BMC Pregnancy Childbirth. 2017;17(1):1–11.
23. Das A, Nawal D, Singh MK, Karthick M, Pahwa P, Shah MB, et al. Impact of a Nursing Skill-Improvement Intervention on Newborn-Specific Delivery Practices: An Experience

from Bihar, India. Birth. 2016;43(4):328–35.
24. Bellad RM, Bang A, Carlo WA, McClure EM, Meleth S, Goco N, et al. A pre-post study of a multi-country scale up of resuscitation training of facility birth attendants: Does Helping Babies Breathe training save lives? BMC Pregnancy Childbirth. 2016;16(1):1–10.
25. International Institute for Population Sciences. National Family Health Survey-4. State Fact Sheet Bihar 2015-16. 2015; Available from: http://rchiips.org/nfhs/factsheet_NFHS-4.shtml
26. CARE India. Integrated Family Health Initiative: Catalysing change for healthy communities [Internet]. 2012. Available from: http://www.care.org/sites/default/files/documents/MH-2013-BIHAR_IFHI_Program Summary.pdf
27. PRONTO International. ABOUT PRONTOPack™ [Internet]. [cited 2018 Apr 25]. Available from: http://prontointernational.org/our-resources/simulation-supplies/about-prontopack/
28. Stata 14 | Stata [Internet]. [cited 2018 Feb 15]. Available from: https://www.stata.com/stata14/
29. World Health Organization. Service Availability and Readiness Assessment (SARA) An annual monitoring system for service delivery Reference Manual.
30. Iacus SM, King G, Porro G. Causal inference without balance checking: Coarsened exact matching. Polit Anal. 2012;20(1):1–24.
31. Das J, Hammer J. Which doctor? Combining vignettes and item response to measure clinical competence. J Dev Econ. 2005 Dec;78(2):348–83.
32. Leonard KL, Masatu MC. The use of direct clinician observation and vignettes for health services quality evaluation in developing countries. Soc Sci Med [Internet]. 2005 Nov [cited 2018 Apr 3];61(9):1944–51. Available from: http://www.ncbi.nlm.nih.gov/pubmed/15936863
33. Peabody JW. Measuring the Quality of Physician Practice by Using Clinical Vignettes: A Prospective Validation Study. Ann Intern Med. 2004 Nov;141(10):771.
34. Rushforth HE. Objective structured clinical examination (OSCE): Review of literature and implications for nursing education. Nurse Educ Today [Internet]. 2007 Jul 1;27(5):481–90. Available from: http://dx.doi.org/10.1016/j.nedt.2006.08.009
35. Patrício MF, Julião M, Fareleira F, Carneiro AV. Is the OSCE a feasible tool to assess competencies in undergraduate medical education? Med Teach [Internet]. 2013 Jun 1;35(6):503–14. Available from: https://doi.org/10.3109/0142159X.2013.774330
36. Aimable M, Birgitta E, Cyprien B, Alphonse R, Lars-Åke P. Evaluating Helping Babies Breathe: training for healthcare workers at hospitals in Rwanda. Acta Paediatr [Internet]. 2012 Nov 1;102(1):e34–8. Available from: https://doi.org/10.1111/apa.12034
37. Thukral A, Lockyer J, Bucher SL, Berkelhamer S, Bose C, Deorari A, et al. Evaluation of an educational program for essential newborn care in resource-limited settings: Essential Care for Every Baby. BMC Pediatr [Internet]. 2015;15(1). Available from: http://dx.doi.org/10.1186/s12887-015-0382-z
38. Goudar SS, Dhaded SM, McClure EM, Derman RJ, Patil VD, Mahantshetti NS, et al. ENC training reduces perinatal mortality in Karnataka, India. J Matern Neonatal Med [Internet]. 2012 Jun 27 [cited 2018 Apr 2];25(6):568–74. Available from: http://www.tandfonline.com/doi/full/10.3109/14767058.2011.584088
39. Varghese B, Krishnamurthy J, Correia B, Panigrahi R, Washington M, Ponnuswamy V, et al. Limited Effectiveness of a Skills and Drills Intervention to Improve Emergency Obstetric and Newborn Care in Karnataka, India: A Proof-of-Concept Study. Glob Heal Sci Pract [Internet]. 2016 [cited 2018 Apr 30];4(4). Available from:

For peer review only

https://www.ncbi.nlm.nih.gov/pmc/articles/PMC5199176/pdf/582.pdf
40. Bang A, Patel A, Bellad R, Gisore P, Goudar SS, Esamai F, et al. Helping Babies Breathe (HBB) training: What happens to knowledge and skills over time? [cited 2018 Apr 2]; Available from: https://bmcpregnancychildbirth.biomedcentral.com/track/pdf/10.1186/s12884-016-1141-3
41. Das J, Mohpal A. Socioeconomic Status And Quality Of Care In Rural India: New Evidence From Provider And Household Surveys. Health Aff (Millwood) [Internet]. 2016;35(10):1764–73. Available from: http://content.healthaffairs.org/content/35/10/1764%5Cnhttp://content.healthaffairs.org/1340_reprints.php%5Cnhttp://content.healthaffairs.org/subscriptions/etoc.dtl%5Cnhttps://fulfillment.healthaffairs.org%5Cnhttp://content.healthaffairs.org/%5Cnhttp://ww
42. Das J, Hammer J. Quality of Primary Care in Low-Income Countries: Facts and Economics. Wws-RoxenPrincetonEdu [Internet]. 1:1–41. Available from: http://wws-roxen.princeton.edu/chwpapers/papers/Das_Hammer_AnnualReviewEconomics_FinalRevision.pdf
43. Leonard K, Masatu M. Professionalism and the know-do gap: exploring intrinsic motivation among health workers in Tanzania. Health Econ [Internet]. 2010 Nov 11;19(12):1461–77. Available from: https://doi.org/10.1002/hec.1564

For peer review only

FIGURE LEGENDS

Figure 1: Effect of mentoring on ability of ANMs and Staff Nurses

Figure 2: Trends in nurse ability after completion of mentoring (lowess estimates)

For peer review only

Table 1: Characteristics of matched and unmatched sample

Indicator	Matched PHCs		Total sample*
	Mentored	Comparison	
**Primary Health Centers**			
N (PHC)	134	56	239
Connected to metaled road (%)	92	87	89
Electricity always available (%)	90	89	86
Labor room has functional handwashing	100	100	93
Oxytocin available	83	75	79
Number of beds	10.9 (10.95)	10.2 (14.44)	15.3 (30.19)
Number of deliveries in past year	193.4 (93.91)	133.8 (93.39)	170.6 (101.11)
**Nurses**			
N(staff-nurses & ANMs)	265	112	474
Completed full course of AMANAT mentoring (%)	93	-	-
ANMs (%)	89	87	84
Regular staff (%)	63	71	67
Age	41.1 (8.75)	43.2 (9.70)	41.8 (9.22)

*Total sample includes the pool of mentored and comparison PHCs/nurses.

For peer review only

Table 2: Overall ability of staff nurse and auxiliary nurse-midwife (ANM) to manage normal and complicated deliveries

	Normal Delivery		Post-Partum Hemorrhage		Neonatal Resuscitation	
	M	C	M	C	M	C
**Auxiliary Nurse Midwife (ANM)**						
N (ANMs)	237	98	237	98	237	98
n (items)	11850	4493	4266	1617	3792	1438
% of correct actions (unadjusted)	53	36	49	24	53	25
Predicted probability of correct action	0.53	0.36	0.50	0.25	0.53	0.24
95% CI	[0.51,0.55]	[0.33,0.38]	[0.48,0.53]	[0.22,0.28]	[0.49,0.56]	[0.19,0.30]
**Staff-nurse**						
N (staff nurse)	28	14	28	14	28	14
n (items)	1400	1108	504	399	448	354
% of correct actions (unadjusted)	55	41	48	33	58	36
Predicted probability of correct action	0.55	0.42	0.50	0.37	0.57	0.38
95% CI	[0.51,0.59]	[0.37,0.48]	[0.44,0.56]	[0.31,0.42]	[0.49,0.66]	[0.31,0.46]

Note: Predicted probabilities are the probability that a staff-nurse/ANM provide a correct action, with all other variables in the regression at their reference value. Multiplying these figures by 100 gives the percentage of correct actions provided by the staff-nurse/ANM.
M: Mentored; C: Comparison

For peer review only

Table 3: Correct actions provided by auxiliary nurse-midwives (ANM) and staff-nurses by case domains.

	Normal Delivery		Post-Partum Hemorrhage		Neonatal Resuscitation	
	M	C	M	C	M	C
**Auxiliary Nurse Midwife (ANM), % (items)**						
N(ANMs)	237	98	237	98	237	98
Correct history & examination	54* (5,925)	41 (2,246)	63* (1,422)	34 (539)	55* (474)	28 (180)
Correct case identification	96 (237)	99 (90)	84* (237)	51 (90)	83* (237)	50 (90)
Correct case management actions	51* (5,688)	28 (2,156)	37* (2,607)	16 (988)	51* (3,081)	22 (1,168)
**Staff nurses, % (items)**						
N (staff nurses)	28	14	28	14	28	14
Correct history & examination	55* (700)	41 (554)	63* (168)	46 (133)	48 (56)	28 (44)
Correct case identification	96 (28)	100 (22)	89 (28)	76 (22)	100* (28)	75 (22)
Correct case management actions	54* (672)	38 (531)	38* (308)	22 (244)	56* (364)	34 (288)

*Chi-sq test p-value<0.05; M: Mentored; C: Comparison

For peer review only

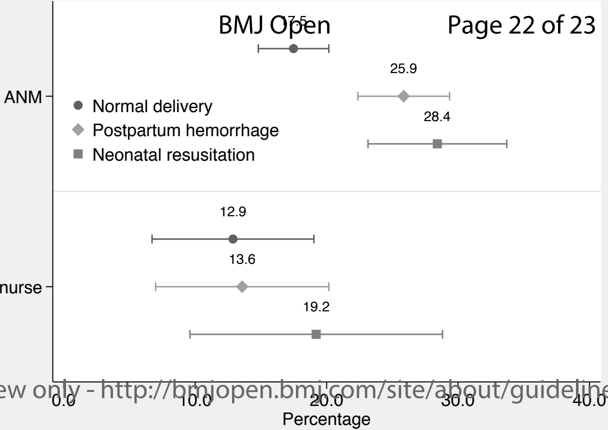

er review only - http://bmjopen.bmj.com/site/about/guidelines

Note: Figures show the percentage point difference (and 95% CI) in quality actions undertaken between the mentored and non-mentored group adjusted for other variables

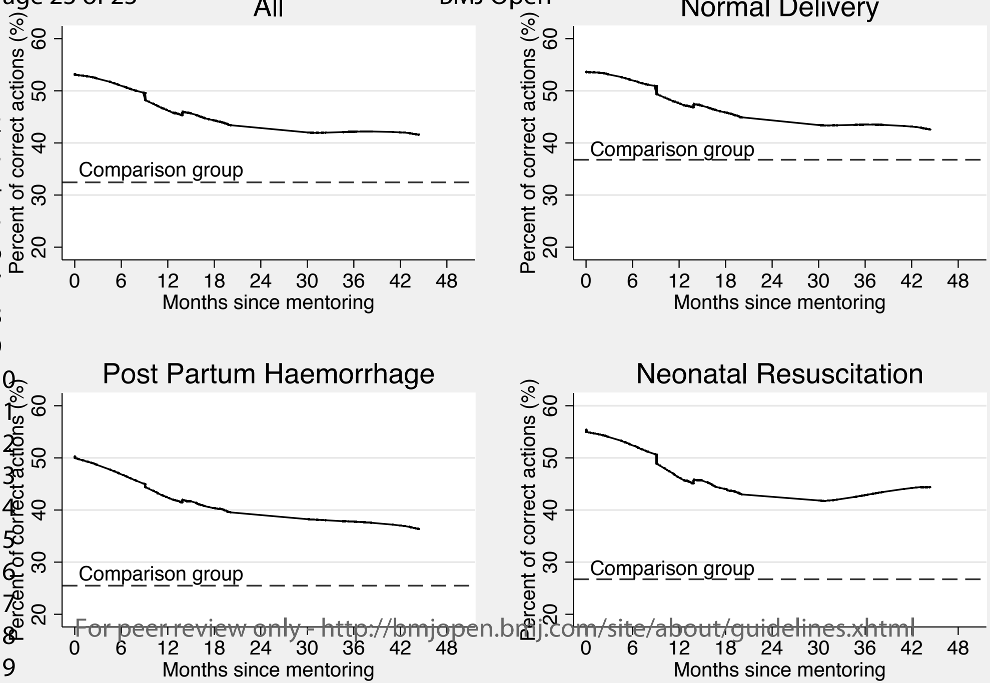

For peer review only - http://bmjopen.bmj.com/site/about/guidelines.xhtml

BMJ Open

Where there is no nurse – an observational study of large-scale mentoring of auxiliary nurses to improve quality of intrapartum care at primary health centers in India.

Journal:	*BMJ Open*
Manuscript ID	bmjopen-2018-027147.R1
Article Type:	Research
Date Submitted by the Author:	08-Feb-2019

Complete List of Authors:	Rao, Krishna; Johns Hopkins University Bloomberg School of Public Health, Department of International Health<br>Srivastava, Swati; Johns Hopkins University Bloomberg School of Public Health, International Health<br>Warren, Nicole; Johns Hopkins University School of Nursing<br>Mayra, Kaveri; Oxford Policy Management<br>Gore, Aboli; CARE India Solutions for Sustainable Development<br>Das, Aritra; CARE India Solutions for Sustainable Development<br>Ahmed, Saifuddin; Johns Hopkins University Bloomberg School of Public Health, Population, Family And Reproductive Health

**Primary Subject Heading**:	Global health
Secondary Subject Heading:	Health policy, Health services research, Obstetrics and gynaecology, Public health
Keywords:	Quality in health care < HEALTH SERVICES ADMINISTRATION & MANAGEMENT, Health policy < HEALTH SERVICES ADMINISTRATION & MANAGEMENT, OBSTETRICS, PRIMARY CARE

SCHOLARONE™
Manuscripts

Title: Where there is no nurse – an observational study of large-scale mentoring of auxiliary nurses to improve quality of intrapartum care at primary health centers in India.

Authors: Krishna D. Rao[1*], Swati Srivastava[2], Nicole Warren[3], Kaveri Mayra[4] , Aboli Gore[5], Aritra Das[5], Saifuddin Ahmed[6]

[1]Department of International Health, Johns Hopkins Bloomberg School of Public Health, Baltimore, Maryland, United States of America

[2]Division Health Economics Health Financing, Heidelberg Institute of Global Health, Medical Faculty and University Hospital, Heidelberg University, Heidelberg, Germany.

[3] Johns Hopkins School of Nursing, Baltimore, Maryland, United States of America

[4] Oxford Policy Management, New Delhi, India

[5] CARE India Solutions for Sustainable Development, H No. 14, Patliputra Colony, Patna, Bihar, PIN Code— 800013, India

[6] Department of Population, Family and Reproductive Health, Johns Hopkins Bloomberg School of Public Health, Baltimore, Maryland, United States of America

[*]Corresponding author
Postal Address: Suite E-8148, Department of International Health, Johns Hopkins University, 315 N. Wolfe Street, Baltimore, MD 21215.
Email: kdrao@jhu.edu
Telephone Number: 443-432-9551

Word Count: 6,091 words

For peer review only

Abstract:

Objective: Scarcity of trained clinicians in LMICs often results in de-facto shifting of clinical tasks from higher to lower cadres; this raises concerns about the quality of care. This study examines if a long-term mentoring program improved the ability of auxiliary nurse-midwives (ANMs), who function as paramedical community health workers, to provide quality obstetric care, how they compared with staff nurses, and if these gains were sustained.
Design: Quasi-experimental post-test with matched comparison group. Follow-up surveys of nurses.
Setting: Primary health centers (PHC) in the state of Bihar, India; a total of 239 PHCs surveyed and matched analysis based on 190 (134 intervention and 56 comparison) facilities.
Participants: Analysis based on 335 ANMs (237 mentored and 98 comparison) and 42 staff nurses (28 mentored and 14 comparison).
Intervention: Large-scale mentoring program for a duration of six to nine months focused on ANMs and staff nurses at PHCs with the aim of improving the quality of basic emergency obstetric and newborn care.
Primary outcome measures: Nurse ability to provide correct actions in managing cases of normal delivery, postpartum hemorrhage, and neonatal resuscitation assessed using a combination of clinical vignettes and Objective Structured Clinical Examinations.
Results: Mentoring improved correct actions taken by ANMs to manage normal deliveries by 17.5 (95% CI: 14.8, 20.2), post-partum hemorrhage by 25.9 (95% CI: 22.4, 29.4), and neonatal resuscitation 28.4(95% CI: 23.2, 33.7) percentage points. There was no significant difference between the average ability of mentored ANMs and staff nurses. However, they provided only half the required correct actions. Ability was highest in the period immediately after mentoring and declined subsequently.
Discussion: Mentoring programs can improve the ability of ANMs to provide quality intrapartum care at levels comparable with trained nurses. However, remaining quality gaps and loss of ability gains over time make continuing education programs critical.

Strengths and limitations of the study

- We were able to assess if the quality of obstetric care produced by auxiliary nurse-midwives (ANM), who typically serve as paramedical workers in rural communities in India, can be improved through on-site mentoring to levels comparable with trained staff nurses.
- This was a large scale on-site mentoring program that was implemented in around 340 (60%) primary care facilities and included both ANMs and staff nurses in the state of Bihar.
- Nurse ability measured using a unique tool that captures both knowledge and demonstrated skill.
- Observational study includes both intervention (mentored) and comparison (non-mentored) ANMs; however, no pre-intervention observations on nurse ability, but staff nurses serve as reference standard.
- ANM and staff nurse demonstrated ability to provide quality care can be different from what they do in actual practice.

For peer review only

INTRODUCTION

Access to a skilled birth attendant is a key strategy to reduce maternal and neonatal deaths in low and middle-income countries. India's high maternal mortality of 130 per 100,000 live births contributes 19% of global maternal deaths[1,2]. Institutional deliveries have been actively promoted by the government to increase access to skilled birth attendants; for example, since 2005 the government has offered cash incentives to pregnant women (and health workers at public facilities) if they deliver at a health facility. This has resulted in the institutional delivery rate doubling between 2005 and 2014 (from 39% to 79%)[3]. Notably, around 52% (54% in rural) of the deliveries nationwide occurred at a public sector facility; this highlights the prominent role of the public sector in providing women access to skilled birth attendants[3].

The remarkable increases in institutional deliveries in India have also raised concern about the quality of obstetric care. Studies have pointed out that despite substantial increases in institutional deliveries there has not been a concomitant reduction in maternal mortality, and its effect on neonatal mortality is unclear [4–10]. While the infrastructure and resources available at health facilities have been an ongoing challenge, more recently, attention has also focused on the performance of health care providers[11-14]. A recent study of nurses' management of obstetric complications at public facilities in India found that only 14% of nurses conducted initial assessments, 58% made a correct clinical diagnosis, and 20% provided first-line care[14]. A variety of factors constrain the ability of nurses to provide quality care – inadequate infrastructure, poor training, limited access to continuing education and professional support from experienced clinicians, lack of agency in the workplace, and entrenched detrimental clinical practices[6,15–18].

In health system contexts where trained clinicians are scarce, clinical functions are often shifted to health workers with less training and without appropriate support, which raises concerns about the quality of care [19]. In the state of Bihar where this study is situated, the lack of doctors and staff nurses at primary health centers (PHC) has resulted in lesser trained Auxiliary Nurse-midwives (ANM) providing obstetric care. Both staff nurses and ANMs are cadres within the public sector health system. Staff nurses have finished high school and have completed a diploma in General Nursing and Midwifery (GNM), a three year course in basic nursing [20]. GNM nurses are trained to conduct deliveries and manage complications. ANMs have completed high school and possess a two year diploma that trains them to be multi-purpose community health workers; their course work includes some training in managing deliveries [20]. Discussions the authors had with ANMs in Bihar indicate that their diploma programs include some course work but no practical training in managing deliveries. The curriculum of GNM and ANM programs is set by the Nursing Council of India [20]. ANMs were initially introduced into the health workforce in the 1950s to serve primarily as midwives at PHCs and sub-centers[21,22]. However, overtime their function changed from that of a midwife to a paramedical health worker who provides a range of services such as family planning, immunizations and some level of antenatal care[21,22]. In Bihar, because staff nurses are scarce at PHCs, ANMs have taken on the responsibility of managing deliveries.

Efforts to improve clinical skills of in-service nurses[1] have traditionally taken the form of short duration training sessions, often away from the health facility, which are not very effective for skill building. On-site clinical mentoring programs, which involve longer term engagement and relationship building between mentor and mentee, have shown to be effective in improving nurse knowledge and skills around essential obstetric and neonatal care in other countries, and

[1] We use the term nurses to include both ANMs and staff nurses.

For peer review only - http://bmjopen.bmj.com/site/about/guidelines.xhtml

more recently in India [23-27]. When mentors become part of the mentee's environment, mentoring functions such as didactic instruction, demonstrations, and support and encouragement, have the potential to improve mentee skills and self-efficacy to perform effectively and overcome challenges. On-site mentoring programs for nurses at PHCs in Bihar report increased nurse knowledge and management of normal and complicated deliveries, increased adherence to quality protocols during delivery, and increased confidence [26,27]. In other contexts mentoring was associated with a reduction in mortality for low birth weight babies[28].

This paper reports on a large-scale mentoring program to improve the quality of intrapartum care produced by ANMs (and staff nurses) at PHCs in the state of Bihar, India. Specifically, this study investigates if mentoring improved the ability of ANMs to manage normal and complicated deliveries; how mentored ANMs and staff nurses compared in ability, and the sustainability of the mentoring effect. Nurse ability was assessed in managing standardized cases of normal delivery, postpartum hemorrhage, and neonatal resuscitation. These cases were selected because mentees were taught to manage these conditions; hemorrhage is a leading cause of maternal mortality, and birth asphyxia a leading cause of neonatal mortality in India [29,30].

The state of Bihar (population 100 million) is among India's resource poor states. PHCs provide outpatient and occasionally inpatient care, maternal health services, family planning, public health services, and basic diagnostic and laboratory services. They cover between 20,000 to 30,000 people and are staffed with a doctor, nurses, and other supporting staff. PHCs are central to facility-based delivery of obstetric services because they are the first level in the public system which has clinicians trained to provide obstetric care such as doctors and nurses. In 2015-16, nearly 64% of all births were in health facilities and 36% at home; urban areas had 74% institutional births while rural areas had 63%[31]. Institutional births in public facilities have increased from just 4% of all institutional births in 2005-06 to almost 48% in 2015-16[31].

AMANAT nurse mentoring program in Bihar

CARE-India in collaboration with the Government of Bihar implemented the AMANAT on-site nurse mentoring program between March 2015 to January 2017. AMANAT aimed to improve the quality of basic emergency obstetric and newborn care (BEmONC) at primary health care facilities in the state of Bihar [32,33]. Because the nomenclature of primary care facilities is somewhat ambiguous in Bihar and change over time, we refer to AMANAT health facilities as PHCs in this study. The AMANAT program was preceded by a pilot from 2011 to 2013 in eight districts of Bihar [34]. The AMANAT program was implemented in a staggered manner over four phases; the program content was similar across phases [33]. In each phase 80 PHCs were purposively selected by CARE-India for mentoring. Several criteria determined selection of a PHC– availability of nurses or ANMs at the PHCs, the volume of deliveries, the infrastructural readiness of the PHC, the willingness of the PHC management to undertake mentoring, and the proximity of the PHC to other PHCs in the area such that mobile mentoring teams could easily rotate between them. In our analysis we include many of these selection criteria to construct a counterfactual set of health facilities. In total 400 out of 534 block-level (the smallest administrative unit within a district) PHCs in Bihar received the nurse mentoring intervention.

Prior to mentoring commencing at a PHC, ANMs and staff nurses affiliated with the particular PHC who were willing to participate in the mentoring were identified by the Medical Officer in-charge and requested to be present for the duration of mentoring. On average there were six to eight ANMs/staff nurses mentored in a PHC. In the AMANAT program, the same pair of

For peer review only - http://bmjopen.bmj.com/site/about/guidelines.xhtml

nurse-mentors rotated multiple times through four PHCs over a six to nine-month period, spending one consecutive week at a PHC when they visited (with an average of four PHCs for each nurse-mentor pair) [32]. The nurse-mentors were hired by CARE-Bihar, were recruited from across India, and had a BSc degree in nursing. The nurse-mentors were trained by CARE and provided six days of training on simulation facilitation, team building, communication skills, and debriefing skills, followed by a four-day refresher training around three months into the mentoring period [35].

During their visit to the PHC, the nurse-mentors used structured learning sessions to provide didactic instruction covering a range of topics and bedside mentoring related to managing normal and complicated deliveries [32]. Nurses were trained on basic nursing procedures, infection prevention, basic obstetric and neonatal practices, management of complications such as post-partum hemorrhage, birth asphyxia, pre-eclampsia and others, documentation and reporting, team rapport and communication [32,33,35]. The typical sequence of mentoring sessions was - team building in the first week, followed by week long sessions on normal delivery and immediate newborn care, neonatal resuscitation and post-partum hemorrhage, and repeated mentoring on these conditions along with other obstetric and neonatal emergencies in the remaining weeks [33]. Tailored, structured simulations were used for normal deliveries and complications management through the PRONTOPack and other simulation kits[35-37]. These kits include MamaNatalie, a birth simulator worn by a demonstrator to resemble a pregnant woman and used for obstetric practices, and NeoNatalie, a neonate model used to demonstrate neonatal practices. Importantly, the nurse-mentors worked alongside mentees, observed them and provided instruction by demonstration through the co-management of cases. All mentoring and training materials were in the local Hindi language. Mentoring sessions were conducted in groups. ANMs and staff nurses were not mentored in separate groups. While a set of standard topics were covered during mentoring, certain aspects were emphasized to suit the particular learning needs of mentees and/or respond to clinical scenarios that emerged at the mentoring site.

METHODS

Study design

To assess the effect of mentoring on nurse ability we use a quasi-experimental post-test with matched comparison group design. PHCs exposed to AMANAT mentoring were matched on several criteria with non-mentored PHCs (see section Analytical Methods). The AMANAT program was implemented in four phases, such that, in each phase 80 PHCs were purposively selected. Because the nomenclature of primary care facilities is somewhat ambiguous and can include sub-district hospitals and other types of primary health care facilities, and these designations can change over time, we refer to AMANAT health facilities as PHCs in this study. Of the approximately 534 PHCs in Bihar, 80 PHCs were exposed to mentoring in a pilot phase (2011- 2013) and a further 320 were subsequently exposed during the AMANAT program (2015-2016). Therefore, around 134 PHCs were not exposed to mentoring. This study focuses on the 240 PHCs in phases 2, 3 and 4. These PHCs were first observed twice via repeat cross-sectional surveys – first, just after mentoring was completed and a second survey some months afterwards, depending on the phase. We excluded phase-1 PHCs because they had completed mentoring nearly two years prior to the survey date and we expected significant number of transfers of mentored nurses during this time.

Selection of PHCs: The 240 PHCs in phase 2,3 and 4 of the AMANAT program served as our sampling frame. For Phases 2 and 3, which began mentoring at approximately the same time, 40

For peer review only - http://bmjopen.bmj.com/site/about/guidelines.xhtml

PHCs from each phase were randomly sampled and a total of 79 out of these 80 selected PHCs were surveyed.[2] From Phase 4, 80 PHCS were selected and all were surveyed. Thus, a total of 159 mentored PHCs constitute the pool of intervention group PHCs

134 PHCs in Bihar did not receive any mentoring either through the pilot or AMANAT program. Seven of these PHCs reported 15 or less deliveries per month in the state Health Management Information System (HMIS) and were excluded. The remaining 127 PHCs were mapped to assess geographical proximity to mentored facilities. From these PHCs, 80 PHCs that were closest to a mentored PHC – either from the same block or adjacent block as an intervention PHC - were purposively selected. These 80 PHCs form the pool of comparison group PHCs.

Selection of nurses: In the intervention PHCs, only ANMs and staff nurses who had completed mentoring were eligible to participate. For each sampled PHC the mentored staff nurses or ANMs were identified from mentoring rosters. From this list two were randomly selected and were requested to be present on the day the survey team arrived. From the 159 mentored PHCs a total of 314 staff nurses and ANMs were interviewed (out of a target of 318). From the comparison group of PHCs a convenience sample of two nurses were selected from among those present on the day the survey team arrived. From the 80 comparison PHCs 160 staff nurses and ANMs were interviewed (out of a target of 160).

Data collection and questionnaires: Between September 2016 and November 2017 a series of cross-sectional surveys of PHCs was conducted. PHCs and nurses in phases 2, 3 and 4 were first observed soon after mentoring was completed and the followed-up once after that. Phase 2 PHCS were visited 3 and 15 months after mentoring was completed, Phase 3 PHCS were visited 1 and 13 months after mentoring was completed and Phase 4 PHCs were visited just after and 6 months after mentoring was completed. Data was collected using tablets by trained enumerators who had a GNM or higher nursing degree. Enumerators were recruited from other parts of India and trained in Bihar for ten days to two weeks, depending on the particular batch of enumerators, on practices of managing normal and complicated deliveries, and administering the questionnaires.

Nurse ability was assessed using a structured questionnaire that captured both their knowledge and demonstrated skill in managing normal and complicated deliveries (see analytical methods). This questionnaire also included information on their background (age, number of months of AMANAT training completed, ANM or GNM nurse, regular or contract staff). Further, data from a cross-section survey of health facilities was carried out on the service delivery readiness of PHCs in Bihar and was used for matching intervention and comparison PHCs. In the PHCs surveyed information was collected on availability and condition of drugs, supplies, equipment, and building condition.

Ethical approval: Ethical clearance for the study was received from the Centre for Media Studies (New Delhi) Institutional Review Board vide approval number IRB00006230.

Patient and public involvement: We contacted four senior nurses in Bihar and elsewhere to guide development of the protocols and to train data collectors. The data collectors were also trained nurses. Feedback from nurses during pre-testing enabled designing study protocols so that they did not to place undue time burden on respondent nurses and were flexible to demands of their professional work.

[2] One facility in Phase 2 and 3 received the intervention in the initial pilot phase between Jan 2011 and December 2013 and was excluded.

For peer review only - http://bmjopen.bmj.com/site/about/guidelines.xhtml

Analytical methods

Matching health facilities: The intervention PHCs were selected on the basis of several criteria (see Background section). As such, there are likely to be systematic differences in these and other characteristics between intervention and comparison PHCs. Such observed and unobserved group differences can bias estimates of mentoring impact if they are related to the outcome of interest i.e. nurse ability. We adjust for this potential bias by matching intervention and comparison PHCs on several criteria. The health facility survey collected information on the service delivery readiness of PHCs in Bihar. Using the service delivery/readiness domains indicated in the WHO's Service Availability and Readiness Assessment (SARA)[38] as a guide, we identified the following six indicators for matching PHCs: location – connected to metaled road; basic amenities – power supply usually available or made available through a generator, hand washing station with running water available in labor room; drugs – oxytocin present; infrastructure – sanctioned bed strength; service utilization – average number of deliveries per year in 2016 (from HMIS).

We use Coarsened Exact Matching (CEM) to match PHCs in intervention and comparison groups[39]. After matching, 134 (84%) of the 159 AMANAT PHCs were matched with 56 (70%) of the 80 PHCs in the non-mentored group. From these matched PHCs, there were 265 nurses in the intervention group and 112 in the comparison group.

Measuring nurse ability: To measure nurse ability, we developed a tool that captures both their knowledge and demonstrated skill in managing normal and complicated deliveries. Our tool adapts elements of clinical vignettes with Objective Structured Clinical Examinations (OSCEs). Clinical vignettes have been used to measure health worker knowledge in treating specific disease conditions in the context of a simulated consultation[40-42]. Health care providers are presented with standardized cases specific to a disease condition and their knowledge in managing the specific condition is evaluated using checklists. OSCEs are commonly used across nursing and other allied health professions and involve learners rotating through structured, time-limited stations to demonstrate discrete skills[43,44]. OSCEs are used extensively in many countries to assess competencies in obstetric and newborn care, including in India[45-48]. Our tool combined clinical vignettes and OSCEs by evaluating knowledge (via verbal responses) and skills (via demonstration) in managing normal and complicated deliveries. Nurses and ANMs were evaluated in how they would manage three standardized cases - normal birth, neonatal resuscitation, and postpartum hemorrhage. These cases were selected because mentees were taught to manage these conditions; hemorrhage is a leading cause of maternal mortality, and birth asphyxia a leading cause of neonatal mortality in India [29,30]. Tool development was facilitated by CARE program developers, India-based obstetric experts, and international experts in BEmONC training. The same tool was used in all assessments. A panel of three nurses from the USA and India (Delhi and Bihar) assessed the face and content validity (by indicating if a particular item should be included or not) of the tool.

The three cases on which nurses and ANMs were evaluated – normal delivery, post-partum hemorrhage, and neonatal resuscitation – were structured to assess ability in history taking, examinations, case identification/diagnosis, and management. Two interviewers administered each case – interviewer #1 played the role of the pregnant woman and wore a Mama Natalie, and interviewer #2 observed the interaction using a standard checklist. Each case began with the respondent being presented with a situation by interviewer #2 – for example, the normal delivery case is introduced to the nurse/ANM as "*This is Tara [pointing to interviewer #1]. She is pregnant. She has come to see you at the clinic.*" The respondent is then asked about what history questions she will ask Tara, what examinations will be done, and the respondent will

For peer review only - http://bmjopen.bmj.com/site/about/guidelines.xhtml

demonstrate them on the Mama Natalie. To each relevant history question or examination done, the respondent receives a standard response. For instance, if the respondent asks "When did you have your last menstrual period (LMP)?" a standard response of "nine months" is offered or if the respondent demonstrates checking for fetal heart rate a response of "130" is given. No responses outside of the pre-structured conversation are given. Based on the results of the history and examinations, the nurse is then asked what the diagnosis/clinical decision is (e.g. normal or complicated delivery), and then to demonstrate on the Mama Natalie how she would manage the delivery. Each case comprises a set of items that correspond to correct actions in the domains of history & examinations, case detection/diagnosis, and case management. All relevant correct actions performed was marked off on a checklist.

Data analysis: Each case comprises a set of items that correspond to correct actions necessary for managing the case. The unit of analysis is a necessary action (verbal or demonstration) provided by the respondent in the course of completing a case. Exploratory data analysis (e.g. histograms) was used to examine the distribution of provider responses across PHCs and nurses. Facilities with extreme values were identified and scrutinized. We then computed the proportion of relevant correct actions in history, examination, case detection/diagnosis, and management sections for each of the three cases. Next, using logistic regressions, for each case, we modelled the probability of a nurse providing a correct action as a function of mentoring status, nurse characteristics (GNM nurse (ref: ANM), age, permanent employee (ref: contractual), facility characteristics (connected to metaled road, regular power supply available or made available through a generator, hand washing station with running water available in labor room, oxytocin present; sanctioned bed strength, average number of deliveries per year in 2016, and item dummy variables. Interactions between nurse type and mentoring status were included. Regression standard errors were adjusted for clustering. Goodness of fit test was performed on the final model by inspecting plots of predicted versus observed values. Residual plots were examined to identify outliers and influential observations. Regression results are presented in terms of marginal effects. In reporting study results we followed STROBE guidelines for cross-sectional studies [49]. The statistical analysis was conducted using Stata 14 [50].

RESULTS

Table 1 shows the characteristics of the mentored and comparison PHCs and nurses in the matched and full sample. The full sample (see Total sample) includes the 239 mentored and comparison PHCs and 474 nurses before matching was carried out. After matching there were 134 mentored and 56 comparison PHCs, which yielded 256 mentored and 112 comparison nurses. The matched mentored and comparison PHCs are broadly similar in their characteristics indicating that matching was successful. However, mentored PHCs conducted more deliveries on average than the comparison ones. The PHCs and nurses in the full sample (i.e. Total sample in Table 1), are also broadly similar to the PHCs included in the matched sample in terms of PHC and nurse characteristics, though the matched PHCs have slightly higher values on all the PHC level indicators.

[Table 1 here]

There are several notable points about the characteristics of nurses. First, the overwhelming majority of nurses were ANMs; staff nurses were a minority. The mentored group had a higher proportion of ANMs relative to the comparison group. Secondly, the majority of nurses were regular staff (as opposed to contractual staff); their proportion is higher in the comparison

For peer review only - http://bmjopen.bmj.com/site/about/guidelines.xhtml

group. Finally, nurses across groups are similar in average age. Nurse age is also correlated with service duration; nurses in the mentored and comparison groups had similar practice durations.

Nurses in the AMANAT program typically experienced six full months of mentoring. Overall 93% of nurses reported having at least six months of mentoring exposure (Table 1). Duration of mentoring ranged from two to nine months, with an average of seven months of mentoring. These results suggest that there was high adherence to the AMANAT program among those who participated.

ANM and Staff nurse ability

In all three cases mentored ANMs and staff nurses provided significantly more correct actions than the comparison group (Table 2). For example, mentored (comparison) ANMs provided 53% (36%) correct actions for the normal delivery case, a percentage point difference of 17 points; 49% (24%) for the post-partum hemorrhage case, a percentage point difference of 29 points, and 53% (25%) for the neonatal resuscitation case, a percentage point difference of 28 points. However, the overall proportion of correct actions among mentored ANMs and staff nurses does not exceed 60% in any case. Table 2 also shows the predicted probabilities from the regressions with respondent and PHC characteristics at their reference values. For example, for the normal delivery case mentored (comparison) ANMs provided 53% (36%) of the correct actions, while mentored staff nurses (comparison) provided 55% (42%) correct actions. Notably, the 95% confidence intervals between mentored and comparison nurses do not overlap - mentored ANMs and staff nurses had significantly higher percentage of correct actions compared to the comparison group.

[Table 2 here]

These overall trends are mirrored in staff nurse and ANM performance across the ability domains (Table 3). Mentored ANMs and staff nurses demonstrated significantly more correct actions for history & examinations, and case management relative to those in the comparison group. Mentored (comparison) ANMs provided 54% (41%) of the correct history and examination actions, 63% (34%) for post-partum hemorrhage and 55% (28%) for neonatal resuscitation. Similarly, mentored nurses performed better than the comparison group – mentored (comparison) ANMs provided 51% (28%) of correct case management actions for normal delivery, 37% (16%) for post-partum hemorrhage, and 51% (22%) for neonatal resuscitation. These patterns are also seen in the performance of staff nurses.

[Table 3 here]

The majority of ANMs and staff nurses were able to correctly identify cases (except for ANMs in the comparison group for neonatal resuscitation case) (Table 3). Expectedly, the percent of correct responses was highest for the normal delivery case since the pre-service training of both ANMs and staff nurses include the basics of managing normal delivery. However, the ability to correctly identify a case was higher among mentored nurses (Table 3). Mentored (comparison) ANMs correctly identified 96% (99%) of the normal delivery cases, 84% (51%) of the post-partum hemorrhage case, and 83% (50%) of the neonatal resuscitation case. Similarly, mentored staff nurses had higher case identification than the comparison group except for the normal delivery case. In general, staff nurses did much better than ANMs in correctly identifying a particular case.

For peer review only - http://bmjopen.bmj.com/site/about/guidelines.xhtml

Of interest is to see how well ANMs compare with staff nurses in their ability to manage normal and complicated deliveries. Comparison group (i.e. those without any mentoring) ANMs had significantly lower ability relative to staff nurses for all three cases. However, ANMs that experienced mentoring had similar levels of performance as staff nurses on all three cases. For example, for the normal delivery case mentored ANMs (staff nurses) provided 53% (55%) correct actions, 50% (50%) for post-partum hemorrhage, and 53% (57%) for neonatal resuscitation (Table 2). The overlapping confidence intervals between mentored ANMs and staff nurses indicates that there are no statistically significant differences between them.

Effect of mentoring

Mentored ANMs and staff nurses had significantly greater proportion, relative to the comparison group, of correct actions overall, and for the domains of history & examinations, case identification (except for normal delivery), and in case management (Table 2, 3). Figure 1 illustrates the adjusted mentoring effect (i.e. treatment effect on the treated) on ANMs and staff nurses. These are marginal effects from the regression models with other variables at their reference values. ANMs in the mentored group had 17.5% more correct actions for normal delivery relative to the comparison group, 25.9% more for post-partum hemorrhage, and 28.4% more for neonatal resuscitation. Staff nurses in the mentored group also show similar levels of improved performance. Notably, in all cases the mentoring effect is statistically significant i.e. the 95% confidence intervals do not overlap zero.

[Figure 1 here]

Both ANMs and staff nurses appear to have gained from mentoring, but ANMs have gained more. As seen in Figure 1, the mentoring effect for each case is greater for ANMs than staff nurses. Finally, because no differences in the proportion of correct actions are seen between mentored ANMs and staff nurses (Table 2), it appears that mentoring brought ANMs on par with staff nurses in the management of normal deliveries, post-partum hemorrhage, and neonatal resuscitation.

Of interest is to know if the mentoring effect on nurse ability changes with time. Figure 2 illustrates this by plotting estimates from local area regressions within the matched sample of PHCs; it shows the probability of nurses demonstrating a correct action at different points in time after mentoring was completed. Note that the same nurses are not observed at each point in time. In all three cases, nurse ability declined with time. In the normal delivery case, just after mentoring nurses were able to provide 53% of the correct actions, while nurses observed 43 months (the observation farthest from completion of mentoring) after mentoring were able to provide 43% of correct actions, a ten-percentage point drop. Similar trends are seen in the case of neonatal resuscitation and postpartum hemorrhage. In the neonatal resuscitation case, the slight increase seen at the tail end appears to be an artifact of the data. For this case, nurses just after mentoring were able to provide 55% of the correct actions, which declined to 44% after 43 months post mentoring. For post-partum hemorrhage, just after mentoring nurses were able to provide 50% of correct actions, which fell to 37% 43 months post mentoring, a drop of 13 percentage points.

[Figure 2 here]

For peer review only - http://bmjopen.bmj.com/site/about/guidelines.xhtml

For peer review only

DISCUSSION

In India's public sector health system ANMs function as community based paramedical health workers and provide a range of services such as family planning, immunizations and antenatal care[21,22]. In human resource scarce states like Bihar, where there is a paucity of trained doctors and staff nurses, ANMs are the de-facto providers of obstetric care at many primary care facilities even though they are not formally trained for this role. In our study, in the 239 PHCs surveyed, staff nurses comprised only 16% of the sampled nurses. In a policy environment that aggressively promotes institutional deliveries the quality of care provided by ANMs is an important concern.

A principal finding from this study is that mentoring improved the average ability of ANMs and staff nurses at PHCs to manage normal and complicated deliveries. In all three cases – normal delivery, postpartum hemorrhage, and neonatal resuscitation – mentored ANMs and staff nurses had better average ability compared to non-mentored ones. This finding is consistent with evaluations of mentoring programs in other contexts and India [24-27]. Mentored ANMs (staff nurses) were able to provide 17% (13%) more correct actions for managing normal deliveries, 26% (14%) more for postpartum hemorrhage, and 28% (19%) more for neonatal resuscitation, compared to their non-mentored colleagues. The gains were greater for ANMs since they started from a lower level of ability relative to staff nurses. Among these three cases, for both ANMs and staff nurses the largest gain in ability was in neonatal resuscitation, followed by postpartum hemorrhage, and normal delivery. This is along expected lines since both ANMs and staff nurses are most familiar with the management of normal deliveries, so the incremental effect of mentoring would be relatively smaller. Further, the AMANAT mentoring program emphasized management of complications such as postpartum hemorrhage and neonatal resuscitation, which explains the gains observed in this area.

ANMs that had completed mentoring had the same average ability as staff nurses to manage normal and complicated deliveries. Further, un-mentored ANMs had significantly poorer ability than un-mentored staff nurses. This mentored ANM achievement is particularly salient because ANMs typically operate as paramedical health workers providing services such as family planning, immunizations and some level of antenatal care. It is unlikely that improvements in ANM ability would be achievable outside a mentoring context. The AMANAT program provided a combination of didactic teaching, supportive supervision, and professional support over a long period of time in the context of a mentor-mentee relationship. To the best of our knowledge there was no difference in the way staff nurses and ANMs were mentored, though the program was focused on ANMs given that they primarily manage deliveries at PHCs.

Mentoring improved case identification among ANMs. The vast majority of mentored ANMs were able to correctly identify a normal delivery case, 84% correctly identified a postpartum hemorrhage case, and 83% the neonatal resuscitation case. While non-mentored ANMs and staff nurses performed similarly as mentored ANMs in correctly identifying normal deliveries, they did quite poorly in identifying the other two cases. For example, among non-mentored ANMs, only half of them were able to correctly identify the postpartum hemorrhage or neonatal resuscitation case. The biggest improvement in correctly identifying a case was among ANMs for postpartum hemorrhage and neonatal resuscitation. The improvements observed in recognizing birth complications is important for identifying women for referral or complication management within the PHC.

For peer review only - http://bmjopen.bmj.com/site/about/guidelines.xhtml

There are several reasons why findings from this study might be generalizable to Bihar and to similar contexts. For one, the mentoring program was large-scale and covered most of the state. PHCs surveyed in the study covered the majority of the intervention PHCs and characteristics of the matched sample of PHCs and nurses aligns well with that of the larger pool.

It is important to be cautious about the extent to which quality of care can be improved by a single mentoring program. Even after six to nine months of mentoring through the AMANAT program there remained considerable scope for improving quality of care. Mentored ANMs and staff nurses were able to provide a little more than half the correct actions on all three cases. This is also reflected in the ability domains of history & examinations and case management on which nurses were evaluated. For example, mentored ANMs (nurses) were able to provide only 51% (54%) of correct actions for case management in the normal delivery case, 37% (38%) in the postpartum hemorrhage case, and 51% (56%) correct actions in the neonatal resuscitation case. Secondly, there will be variation in the ability of both ANMs and staff nurses post mentoring. Therefore, not all mentored ANMs and staff nurses will have the ability to properly manage normal or complicated deliveries. Moreover, the work environment can also affect nurse performance; the availability of adequate infrastructure, adequate staff, the management environment are all factors that can influence performance and modify the effect of mentoring.

Another reason for being cautious is that the mentoring effect on nurse ability appears to decay over time. In all three cases we find that nurse ability was highest in the period immediately after mentoring and this declined with the passage of time. The decay was least for the normal delivery case and most for the neonatal resuscitation case. This has also been observed in other nurse mentoring studies in India, which have documented declines in nurse practice and knowledge[26,51]. These patterns highlight the importance of continuing health worker education and make a strong case for mentoring or similar programs which are embedded within the health system to be offered on a frequent basis to health workers.

A limitation of this study is that we did not observe the ability of nurses before they started mentoring. As such, our estimates of the mentoring effect will be biased if the matched comparison group nurses do not have the same average ability as those in the mentored group before they were mentored. If at baseline comparison nurses had lower ability relative to non-mentored nurse, estimates of the mentoring effect will be upwardly biased. There are several reasons why this may not be the case. One source of bias could arise from differences in nurse characteristics between mentored and comparison groups that are related to their ability. Both mentored and comparison group ANMs (or staff nurses) had the same standard pre-service training and require the same qualifications to be recruited into government service. As such, it is unlikely that the extent or quality of nurse pre-service training differed between groups. Further, in our regression we control for difference in nurse and facility characteristics between groups. As such estimates of the mentoring effect are adjusted for differences in nurse characteristics such as age, years of service, and employee type between mentored and non-mentored nurses.

Nurse ability can vary by the characteristics of the environment where they work. For example, studies have noted that the ability of clinical care providers varies with the economic condition of the community i.e. better-off areas have better quality providers[52]. Similarly, nurses working in a facility located in economically better areas (e.g. close to urban areas) might have better ability. As such, differences in the facility location between the mentored and comparison groups can bias estimates of the mentoring effect on nurse ability. If mentored PHCs were located in more favorable areas then estimates of the mentoring effect will be upwardly biased. To address this we created the comparison group PHCs to be as similar as possible to the mentored PHCs in terms of location and physical environment. Comparison group PHCs were selected such that

For peer review only - http://bmjopen.bmj.com/site/about/guidelines.xhtml

they were in close geographic proximity (from the same or adjacent administrative block) to the mentored PHCs. Further, we matched mentored and comparison PHCs on location, service readiness, and PHC use criteria.

Improved nurse ability need not translate into better practice in managing deliveries. Factors such as motivation, the work environment and support from colleagues, among others, mediate the pathway between ability to practice. However, studies that have examined the association between ability and practice report that these measures are correlated, though there is a typically a gap between what clinicians know and what they do in practice [53]. In general, more able clinicians also "do more" in practice, and ability measures the upper bound of what is possible in practice [53].

Mentoring programs can improve the quality of care of lesser trained health workers like ANMs to levels comparable to those of staff nurses who have higher levels of training. In health systems which struggle to recruit highly trained health workers to underserved areas, such mentoring programs offer a way of making task shifting strategies successful. However, other considerations like program costs and the ability to sustain such programs for long periods are also important for scale-up. To sustain the quality gains from mentoring it is important to expose health workers to regular continuing education programs.

For peer review only

ACKNOWLEDGEMENTS

The authors would like to acknowledge Priya Nanda (BMGF, Delhi), Yamini Atmavilas (BMGF Delhi), Dr. Sridhar Srikantiah (CARE-Bihar), Dr. Tanmay Mahapatra (CARE-Bihar), Tom Newton-Lewis (Oxford Policy Management, Delhi).

COMPETING INTERESTS

The authors declare they have no competing interests.

FUNDING

The study was funded by the Bill and Melinda Gates Foundation (https://www.gatesfoundation.org/) via grant number OPP1142884. The funders had no role in study design, data collection and analysis, decision to publish, or preparation of the manuscript.

AUTHORS CONTRIBUTIONS

The study was conceived and designed by KDR, NW, and SA. Study protocols were developed and by KDR, NW, SS, and KM. Field work was conducted by KDR, NW, SS, and KM. Data analysis was done by KDR, SS and SA. The paper was written primarily by KDR, SS and NW, with contributions from KM, AG, AD and SA.

DATA SHARING

The data contained within this study can be obtained by writing to kdrao@jhu.edu.

For peer review only

REFERENCES

1. Sample Registration System. Special bulletin on maternal mortality in india 2014-16. Registrar General of India 2018. Available from: http://www.censusindia.gov.in/vital_statistics/SRS_Bulletins/MMR%20Bulletin-2014-16.pdf (accessed 3 Feb 2019)
2. World Health Organization. Trends in maternal mortality: 1990 to 2015: estimates by WHO, UNICEF, UNFPA, World Bank Group and the United Nations Population Division. Geneva; 2015. Available from: www.who.int/about/licensing/
3. International Institute for Population Sciences. National Family Health Survey-4 2015-16 – India fact sheet. Mumbai; Available from: http://rchiips.org/nfhs/pdf/NFHS4/India.pdf (accessed Apr 28 2018).
4. Randive B, Diwan V, De Costa A India's Conditional Cash Transfer Programme (the JSY) to Promote Institutional Birth: Is There an Association between Institutional Birth Proportion and Maternal Mortality? PLoS One 2013;8(6).
5. Sabde Y, Diwan V, Randive B, Chaturvedi S, Sidney K, Salazar M, et al. The availability of emergency obstetric care in the context of the JSY cash transfer programme in Madhya Pradesh, India. BMC Pregnancy Childbirth 2016;16.
6. Chaturvedi S, Randive B, Diwan V, De Costa A, Baradaran HR.Quality of Obstetric Referral Services in India's JSY Cash Transfer Programme for Institutional Births: A Study from Madhya Pradesh Province. PLoS One 2014;9(5).
7. Ameh C, Msuya S, Hofman J, Raven J, Mathai M, Van Den Broek N. Status of Emergency Obstetric Care in Six Developing Countries Five Years before the MDG Targets for Maternal and Newborn Health.PlosOne 2018. 2012;7(12)
8. Ng, M, Misra, A, Diwan, V, Agnani, M, Levin-Rector, A, & De Costa, A. An assessment of the impact of the JSY cash transfer program on maternal mortality reduction in Madhya Pradesh, India. *Global health action* 2014; *7*, 24939.
9. Powell-Jackson T, Mazumdar S, Mills A. Financial incentives in health: New evidence from India's Janani Suraksha Yojana. Journal of Health Economics 2015; 43: 154-169.
10. Lim SS, Dandona L, Hoisington JA, James SL, Hogan MC, Gakidou E. India's Janani Suraksha Yojana, a conditional cash transfer programme to increase births in health facilities: an impact evaluation. *Lancet 2010;* 375: 2009-2023
11. Mony PK, Krishnamurthy J, Thomas A, Sankar K, Ramesh BM, Moses S, et al. Availability and Distribution of Emergency Obstetric Care Services in Karnataka State, South India: Access and Equity Considerations. PLoS One 2013; 8(5).
12. Sharma G, Powell-Jackson T, Haldar K, Bradley J, Filippi V. Quality of routine essential care during childbirth: clinical observations of uncomplicated births in Uttar Pradesh, India. Bulletin of the World Health Organization 2017; 95(6):419–29.
13. Jayanna K, Mony P, Bm R, Thomas A, Gaikwad A, Hl M, et al. Assessment of facility readiness and provider preparedness for dealing with postpartum haemorrhage and pre-eclampsia/eclampsia in public and private health facilities of northern Karnataka, India: a cross-sectional study. BMC Pregnancy Childbirth 2014;14:304.
14. Chaturvedi S, Upadhyay S, De Costa A. Competence of birth attendants at providing emergency obstetric care under India's JSY conditional cash transfer program for institutional delivery: an assessment using case vignettes in Madhya Pradesh province. BMC Pregnancy Childbirth 2014; 14:174.
15. Evans CL, Maine D, McCloskey L, Feeley FG, Sanghvi H. Where there is no obstetrician - increasing capacity for emergency obstetric care in rural India: An evaluation of a pilot program to train general doctors. Int J Gynecol Obstet 2009; 107(3):277–82.
16. Iyengar SD, Iyengar K, Martines JC, Dashora K, Deora KK. Childbirth practices in rural

For peer review only - http://bmjopen.bmj.com/site/about/guidelines.xhtml

Rajasthan, India: Implications for neonatal health and survival. J Perinatol 2014; 28(SUPPL. 2):23–30.

17. Iyengar K, Jain M, Thomas S, Dashora K, Liu W, Saini P, et al. Adherence to evidence based care practices for childbirth before and after a quality improvement intervention in health facilities of Rajasthan, India. BMC Pregnancy Childbirth 2014;14(1):1–12.
18. Iyengar K, Iyengar SD. Emergency obstetric care and referral: experience of two midwife-led health centres in rural Rajasthan, India. Reprod Health Matters 2009; 17(33):9–20.
19. Rao KD, Sundararaman T, Bhatnagar A, Gupta G, Kokho P, Jain K. Which doctor for primary health care? Quality of care and non-physician clinicians in India. Soc Sci Med 2013;84:30–4.
20. Indian Nursing Council. Retreived from: http://www.indiannursingcouncil.org/nursing-programs.asp?show=elig-crit.
21. Mavalankar D, Vora KS. The Changing Role of Auxiliary Nurse Midwife (ANM) in India : Implications for Maternal and Child Health. 2008; Indian Institute of Management Ahmedabad, Research and Publication Department, IIMA Working Papers.
22. Malik G. Role of auxiliary nurse midwives in National Rural Health Mission. Nurs J India 2009; 100(4): 88-91.
23. Anatole M, Magge H, Redditt V, Karamaga A, Niyonzima S, Drobac P, et al. Nurse mentorship to improve the quality of health care delivery in rural Rwanda. Nurs Outlook 2013;61(3):137–44.
24. Fischer EA, Jayana K, Cunningham T, Washington M, Mony P, Bradley J, et al. Nurse Mentors to Advance Quality Improvement in Primary Health Centers: Lessons From a Pilot Program in Northern Karnataka, India. Glob Health Sci Pract. 2015 Dec 17;3(4):660-75.
25. Jayanna K, Bradley J, Mony P, Cunningham T, Washington M, Bhat S, et al. Effectiveness of Onsite Nurse Mentoring in Improving Quality of Institutional Births in the Primary Health Centres of High Priority Districts of Karnataka, South India: A Cluster Randomized Trial. PlosOne 2016; 11(9)
26. Das A, Nawal D, Singh MK, Karthick M, Pahwa P, Shah MB, et al. Evaluation of the mobile nurse training (MNT) intervention - a step towards improvement in intrapartum practices in Bihar, India. BMC Pregnancy Childbirth 2017;17(1):1–11.
27. Das A, Nawal D, Singh MK, Karthick M, Pahwa P, Shah MB, et al. Impact of a Nursing Skill-Improvement Intervention on Newborn-Specific Delivery Practices: An Experience from Bihar, India. Birth 2016;43(4):328–35.
28. Bellad RM, Bang A, Carlo WA, McClure EM, Meleth S, Goco N, et al. A pre-post study of a multi-country scale up of resuscitation training of facility birth attendants: Does Helping Babies Breathe training save lives? BMC Pregnancy Childbirth. 2016;16(1):1–10.
29. Montgomery AL, Ram U, Kumar R, Jha P, for The Million Death Study Collaborators. Maternal Mortality in India: Causes and Healthcare Service Use Based on a Nationally Representative Survey. PLoS ONE 2014; 9(1)
30. Million Death Study Collaborators, Bassani, DG, Kumar R, Awasthi S, Morris SK, Paul VK, Shet A, Ram U, Gaffey MF, Black RE, et al. Causes of neonatal and child mortality in India: a nationally representative mortality survey. *Lancet 2010*; 376(9755):1853-60.
31. International Institute for Population Sciences. National Family Health Survey-4. State Fact Sheet Bihar 2015-16. 2015; Available from: http://rchiips.org/nfhs/factsheet_NFHS-4.shtml
32. CARE India. A model for mentorship [Internet]. 2018. Available from http://bihar.care.org/wp-content/uploads/2018/08/Bihar_4_REV_SCREEN.pdf
33. Vail B, Spindler H, Morgan MC, Cohen SR, Christmas A, Sah P, Shah MB, Das A, et al. Care of the mother-infant dyad: a novel approach to conducting and evaluating neonatal

For peer review only - http://bmjopen.bmj.com/site/about/guidelines.xhtml

resuscitation simulation training in Bihar, India. BMC pregnancy and childbirth 2017; *17*(1), 252.

34. CARE India. Integrated Family Health Initiative: Catalysing change for healthy communities [Internet]. 2012. Available from: http://www.care.org/sites/default/files/documents/MH-2013-BIHAR_IFHI_Program Summary.pdf
35. Vail B, Morgan MC, Spindler H, Christmas A, Cohen SR, & Walker DM. The power of practice: simulation training improving the quality of neonatal resuscitation skills in Bihar, India. BMC pediatrics 2018; 18(1), 291.
36. Morgan MC, Dyer J, Abril A, Christmas A, Mahapatra T, Das A, and Walder DM. Barriers and facilitators to the provision of optimal obstetric and neonatal emergency care and to the implementation of simulation-enhanced mentorship in primary care facilities in Bihar, India: a qualitative study. BMC Pregnancy and Childbirth 2018; 18:42
37. Raney, JH, Morgan MC, Christmas A, et al. Simulation-enhanced nurse mentoring to improve preeclampsia and eclampsia care: an education intervention study in Bihar, India. BMC Pregnancy Childbirth 2019; 19: 41.
38. World Health Organization. Service Availability and Readiness Assessment (SARA) An annual monitoring system for service delivery Reference Manual.
39. Iacus SM, King G, Porro G. Causal inference without balance checking: Coarsened exact matching. Polit Anal. 2012;20(1):1–24.
40. Das J, Hammer J. Which doctor? Combining vignettes and item response to measure clinical competence. J Dev Econ. 2005;78(2):348–83.
41. Leonard KL, Masatu MC. The use of direct clinician observation and vignettes for health services quality evaluation in developing countries. Soc Sci Med 2005;61(9):1944–51. A
42. Peabody JW. Measuring the Quality of Physician Practice by Using Clinical Vignettes: A Prospective Validation Study. Ann Intern Med 2004; 141(10):771.
43. Rushforth HE. Objective structured clinical examination (OSCE): Review of literature and implications for nursing education. Nurse Educ Today 2007; 27(5):481–90.
44. Patrício MF, Julião M, Fareleira F, Carneiro AV. Is the OSCE a feasible tool to assess competencies in undergraduate medical education? Med Teach 2013;35(6):503–14.
45. Aimable M, Birgitta E, Cyprien B, Alphonse R, Lars-Åke P. Evaluating Helping Babies Breathe: training for healthcare workers at hospitals in Rwanda. Acta Paediatr 2012;102(1):e34–8.
46. Thukral A, Lockyer J, Bucher SL, Berkelhamer S, Bose C, Deorari A, et al. Evaluation of an educational program for essential newborn care in resource-limited settings: Essential Care for Every Baby. BMC Pediatr 2015;15(1)
47. Goudar SS, Dhaded SM, McClure EM, Derman RJ, Patil VD, Mahantshetti NS, et al. ENC training reduces perinatal mortality in Karnataka, India. J Matern Neonatal Med 2012;25(6):568–74.
48. Varghese B, Krishnamurthy J, Correia B, Panigrahi R, Washington M, Ponnuswamy V, et al. Limited Effectiveness of a Skills and Drills Intervention to Improve Emergency Obstetric and Newborn Care in Karnataka, India: A Proof-of-Concept Study. Glob Heal Sci Pract 2016;4(4).
49. von Elm E1, Altman DG, Egger M, Pocock SJ, Gøtzsche PC, Vandenbroucke JP; STROBE Initiative. The Strengthening the Reporting of Observational Studies in Epidemiology (STROBE) statement: guidelines for reporting observational studies. Bull World Health Organ 2007;85(11):867-72.
50. StataCorp. 2015. *Stata Statistical Software: Release 14*. College Station, TX: StataCorp LP.
51. Bang A, Patel A, Bellad R, Gisore P, Goudar SS, Esamai F, et al. Helping Babies Breathe (HBB) training: What happens to knowledge and skills over time? BMC Pregnancy Childbirth 2016; 16(1):364.

For peer review only - http://bmjopen.bmj.com/site/about/guidelines.xhtml

52. Das J, Mohpal A. Socioeconomic Status And Quality Of Care In Rural India: New Evidence From Provider And Household Surveys. Health Aff 2016;35(10):1764–73.

53. Leonard K, Masatu M. Professionalism and the know-do gap: exploring intrinsic motivation among health workers in Tanzania. Health Econ 2010;19(12):1461–77.

For peer review only

FIGURE LEGENDS

Figure 1: Effect of mentoring on ability of ANMs and Staff nurses

Figure 2: Trends in nurse ability after completion of mentoring (ANM and Staff Nurse combined, lowess estimates)

For peer review only

For peer review only - http://bmjopen.bmj.com/site/about/guidelines.xhtml

Table 1: Characteristics of matched and unmatched sample

**Indicator**	**Matched PHCs**		**Total sample***
	Mentored	Comparison	
Primary Health Centers			
N (PHC)	134	56	239
Connected to metaled road (%)	92	87	89
Electricity always available (%)	90	89	86
Labor room has functional handwashing (%)	100	100	93
Oxytocin available (%)	83	75	79
Average number of beds	10.9 (10.95)	10.2 (14.44)	15.3 (30.19)
Average number of deliveries in past year	193.4 (93.91)	133.8 (93.39)	170.6 (101.11)
Nurses			
N(staff nurses & ANMs)	265	112	474
Completed full course of mentoring (%)	93	-	-
ANMs (%)	89	87	84
Regular (non-contractual) staff (%)	63	71	67
Age (years)	41.1 (8.75)	43.2 (9.70)	41.8 (9.22)

Note: (1) *The 'Total sample' is the pool of mentored (159) and non-mentored (80) PHCs from where the matched sample of mentored (134) and non-mentored (56) PHCs was drawn. Consequently, because some mentored and comparison PHCs were unmatched, the Total Sample does not equal the sum of matched mentored and non-mentored PHCs. (2) Figures in parenthesis are SD.

For peer review only - http://bmjopen.bmj.com/site/about/guidelines.xhtml

For peer review only

Table 2: Overall ability of staff nurse and auxiliary nurse-midwife (ANM) to manage normal and complicated deliveries

	**Normal Delivery**		**Post-Partum Hemorrhage**		**Neonatal Resuscitation**	
	M	C	M	C	M	C
Auxiliary Nurse Midwife (ANM)						
N (ANMs)	237	98	237	98	237	98
n (items)	11850	4493	4266	1617	3792	1438
% of correct actions (unadjusted)	53	36	49	24	53	25
Predicted probability of correct action	0.53	0.36	0.50	0.25	0.53	0.24
95% CI	[0.51,0.55]	[0.33,0.38]	[0.48,0.53]	[0.22,0.28]	[0.49,0.56]	[0.19,0.30]
Staff nurse						
N (staff nurse)	28	14	28	14	28	14
n (items)	1400	1108	504	399	448	354
% of correct actions (unadjusted)	55	41	48	33	58	36
Predicted probability of correct action	0.55	0.42	0.50	0.37	0.57	0.38
95% CI	[0.51,0.59]	[0.37,0.48]	[0.44,0.56]	[0.31,0.42]	[0.49,0.66]	[0.31,0.46]

Note: Predicted probabilities are the probability that a staff nurse/ANM provide a correct action, with all other variables in the regression at their reference value. Multiplying these figures by 100 gives the percentage of correct actions provided by the staff nurse/ANM.
M: Mentored; C: Comparison

For peer review only

Table 3: Correct actions provided by auxiliary nurse-midwives (ANM) and staff nurses by case domains.

	Normal Delivery		Post-Partum Hemorrhage		Neonatal Resuscitation	
	M	C	M	C	M	C
Auxiliary Nurse Midwife (ANM), % (items)						
N(ANMs)	237	98	237	98	237	98
Correct history & examination	54* (5,925)	41 (2,246)	63* (1,422)	34 (539)	55* (474)	28 (180)
Correct case identification	96 (237)	99 (90)	84* (237)	51 (90)	83* (237)	50 (90)
Correct case management actions	51* (5,688)	28 (2,156)	37* (2,607)	16 (988)	51* (3,081)	22 (1,168)
Staff nurses, % (items)						
N (staff nurses)	28	14	28	14	28	14
Correct history & examination	55* (700)	41 (554)	63* (168)	46 (133)	48 (56)	28 (44)
Correct case identification	96 (28)	100 (22)	89 (28)	76 (22)	100* (28)	75 (22)
Correct case management actions	54* (672)	38 (531)	38* (308)	22 (244)	56* (364)	34 (288)

*Chi-sq test p-value<0.05; M: Mentored; C: Comparison

For peer review only

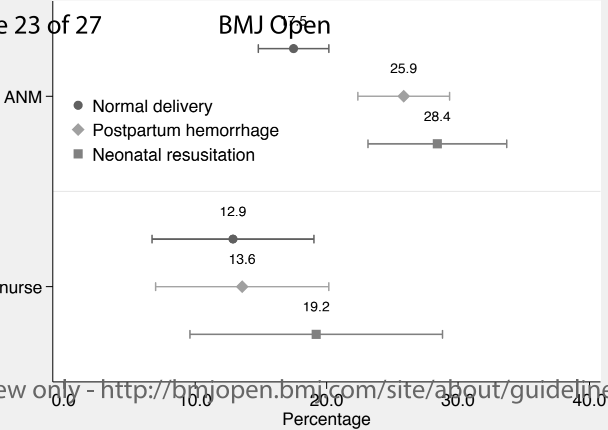

er review only - http://bmjopen.bmj.com/site/about/guidelines

Note: Figures show the percentage point difference (and 95% CI) in quality actions undertaken between the mentored and non-mentored group adjusted for other variables

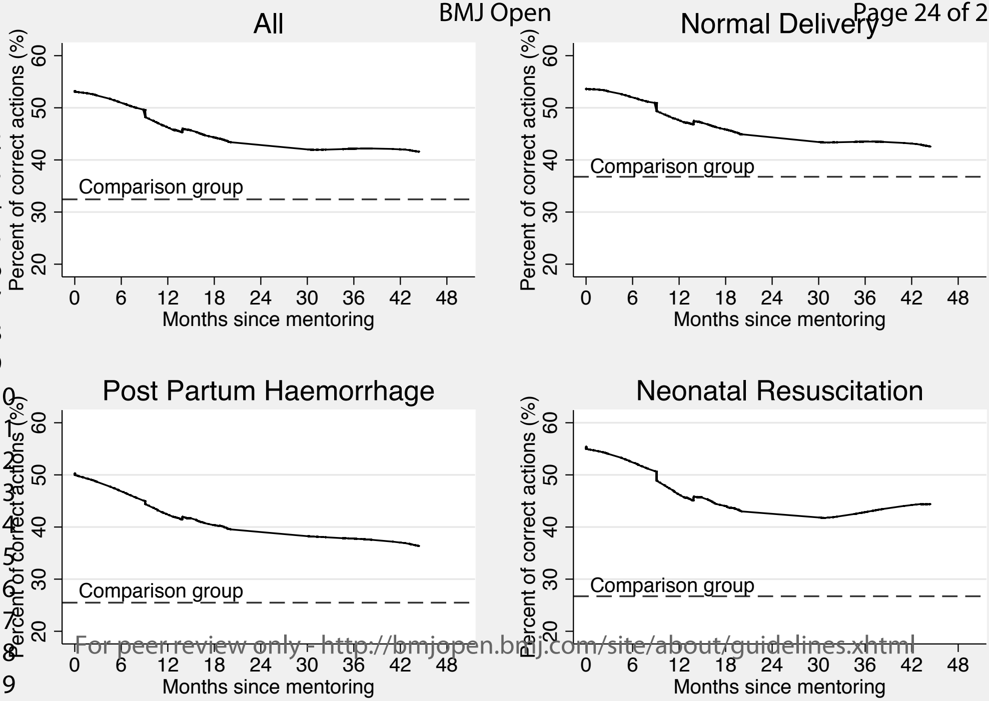

For peer review only - http://bmjopen.bmj.com/site/about/guidelines.xhtml

STROBE 2007 (v4) Statement—Checklist of items that should be included in reports of *cross-sectional studies*

Section/Topic	Item #	Recommendation	Reported on page #/section
**Title and abstract**	1	(*a*) Indicate the study's design with a commonly used term in the title or the abstract	abstract
		(*b*) Provide in the abstract an informative and balanced summary of what was done and what was found	abstract
**Introduction**			
Background/rationale	2	Explain the scientific background and rationale for the investigation being reported	Background, page 1
Objectives	3	State specific objectives, including any prespecified hypotheses	Background, page 2
**Methods**			
Study design	4	Present key elements of study design early in the paper	Methods, page 3
Setting	5	Describe the setting, locations, and relevant dates, including periods of recruitment, exposure, follow-up, and data collection	Background, Page 2; Methods, Page 3&4
Participants	6	(*a*) Give the eligibility criteria, and the sources and methods of selection of participants	Methods, Page 3&4
Variables	7	Clearly define all outcomes, exposures, predictors, potential confounders, and effect modifiers. Give diagnostic criteria, if applicable	Outcome: Methods, Page 4; Exposure: Background, page 1&2
Data sources/ measurement	8*	For each variable of interest, give sources of data and details of methods of assessment (measurement). Describe comparability of assessment methods if there is more than one group	Outcome: Methods, Page 4/5; Other variables Page 4.
Bias	9	Describe any efforts to address potential sources of bias	*Page 4*
Study size	10	Explain how the study size was arrived at	Page 4
Quantitative variables	11	Explain how quantitative variables were handled in the analyses. If applicable, describe which groupings were chosen and why	Page 5
Statistical methods	12	(*a*) Describe all statistical methods, including those used to control for confounding	Page 4 & 5
		(*b*) Describe any methods used to examine subgroups and interactions	Page 5
		(*c*) Explain how missing data were addressed	No missing data

For peer review only

		(*d*) If applicable, describe analytical methods taking account of sampling strategy	N/a
		(*e*) Describe any sensitivity analyses	N/a
**Results**			
Participants	13*	(a) Report numbers of individuals at each stage of study—eg numbers potentially eligible, examined for eligibility, confirmed eligible, included in the study, completing follow-up, and analysed	Page 6
		(b) Give reasons for non-participation at each stage	N/a
		(c) Consider use of a flow diagram	N/a
Descriptive data	14*	(a) Give characteristics of study participants (eg demographic, clinical, social) and information on exposures and potential confounders	Page 6, Table 1
		(b) Indicate number of participants with missing data for each variable of interest	None
Outcome data	15*	Report numbers of outcome events or summary measures	Page 6, Table 2,3
Main results	16	(*a*) Give unadjusted estimates and, if applicable, confounder-adjusted estimates and their precision (eg, 95% confidence interval). Make clear which confounders were adjusted for and why they were included	Table 2, 3; Page 5,6, Figure 1
		(*b*) Report category boundaries when continuous variables were categorized	N/a
		(*c*) If relevant, consider translating estimates of relative risk into absolute risk for a meaningful time period	N/a
Other analyses	17	Report other analyses done—eg analyses of subgroups and interactions, and sensitivity analyses	Interactions Page 6
**Discussion**			
Key results	18	Summarise key results with reference to study objectives	Page 8,9
Limitations	19	Discuss limitations of the study, taking into account sources of potential bias or imprecision. Discuss both direction and magnitude of any potential bias	Page 9
Interpretation	20	Give a cautious overall interpretation of results considering objectives, limitations, multiplicity of analyses, results from similar studies, and other relevant evidence	Page 9 and 10
Generalisability	21	Discuss the generalisability (external validity) of the study results	Page 9
**Other information**			
Funding	22	Give the source of funding and the role of the funders for the present study and, if applicable, for the original study on which the present article is based	Page 11

*Give information separately for cases and controls in case-control studies and, if applicable, for exposed and unexposed groups in cohort and cross-sectional studies.

For peer review only

Note: An Explanation and Elaboration article discusses each checklist item and gives methodological background and published examples of transparent reporting. The STROBE checklist is best used in conjunction with this article (freely available on the Web sites of PLoS Medicine at http://www.plosmedicine.org/, Annals of Internal Medicine at http://www.annals.org/, and Epidemiology at http://www.epidem.com/). Information on the STROBE Initiative is available at www.strobe-statement.org.

For peer review only

Where there is no nurse – an observational study of large-scale mentoring of auxiliary nurses to improve quality of intrapartum care at primary health centers in India.

Journal:	*BMJ Open*
Manuscript ID	bmjopen-2018-027147.R2
Article Type:	Research
Date Submitted by the Author:	01-Jun-2019
Complete List of Authors:	Rao, Krishna; Johns Hopkins University Bloomberg School of Public Health, Department of International Health<br>Srivastava, Swati; Johns Hopkins University Bloomberg School of Public Health, International Health<br>Warren, Nicole; Johns Hopkins University School of Nursing<br>Mayra, Kaveri; Oxford Policy Management<br>Gore, Aboli; CARE India Solutions for Sustainable Development<br>Das, Aritra; CARE India Solutions for Sustainable Development<br>Ahmed, Saifuddin; Johns Hopkins University Bloomberg School of Public Health, Population, Family And Reproductive Health
**Primary Subject Heading**:	Global health
Secondary Subject Heading:	Health policy, Health services research, Obstetrics and gynaecology, Public health
Keywords:	Quality in health care < HEALTH SERVICES ADMINISTRATION & MANAGEMENT, Health policy < HEALTH SERVICES ADMINISTRATION & MANAGEMENT, OBSTETRICS, PRIMARY CARE

SCHOLARONE™ Manuscripts

Title: Where there is no nurse – an observational study of large-scale mentoring of auxiliary nurses to improve quality of intrapartum care at primary health centers in India.

Authors: Krishna D. Rao[1*], Swati Srivastava[2], Nicole Warren[3], Kaveri Mayra[4] , Aboli Gore[5], Aritra Das[5], Saifuddin Ahmed[6]

[1]Department of International Health, Johns Hopkins Bloomberg School of Public Health, Baltimore, Maryland, United States of America

[2]Division Health Economics Health Financing, Heidelberg Institute of Global Health, Medical Faculty and University Hospital, Heidelberg University, Heidelberg, Germany.

[3] Johns Hopkins School of Nursing, Baltimore, Maryland, United States of America

[4] Oxford Policy Management, New Delhi, India

[5] CARE India Solutions for Sustainable Development, H No. 14, Patliputra Colony, Patna, Bihar, PIN Code— 800013, India

[6] Department of Population, Family and Reproductive Health, Johns Hopkins Bloomberg School of Public Health, Baltimore, Maryland, United States of America

[*]Corresponding author
Postal Address: Suite E-8148, Department of International Health, Johns Hopkins University, 315 N. Wolfe Street, Baltimore, MD 21215.
Email: kdrao@jhu.edu
Telephone Number: 443-432-9551

Word Count: 6,091 words

For peer review only

Abstract:

Objective: Clinician scarcity in LMICs often results in de-facto task shifting; this raises concerns about the quality of care. This study examines if a long-term mentoring program improved the ability of auxiliary nurse-midwives (ANMs), who function as paramedical community health workers, to provide quality obstetric care, and how they compared with staff nurses.
Design: Quasi-experimental post-test with matched comparison group.
Setting: Primary health centers (PHC) in the state of Bihar, India; a total of 239 PHCs surveyed and matched analysis based on 190 (134 intervention and 56 comparison) facilities.
Participants: Analysis based on 335 ANMs (237 mentored and 98 comparison) and 42 staff nurses (28 mentored and 14 comparison).
Intervention: Mentoring for a duration of six to nine months focused on nurses at PHCs to improve the quality of basic emergency obstetric and newborn care.
Primary outcome measures: Nurse ability to provide correct actions in managing cases of normal delivery, postpartum hemorrhage, and neonatal resuscitation assessed using a combination of clinical vignettes and Objective Structured Clinical Examinations.
Results: Mentoring increased correct actions taken by ANMs to manage normal deliveries by 17.5 (95% CI: 14.8, 20.2), post-partum hemorrhage by 25.9 (95% CI: 22.4, 29.4), and neonatal resuscitation 28.4(95% CI: 23.2, 33.7) percentage points. There was no significant difference between the average ability of mentored ANMs and staff nurses. However, they provided only half the required correct actions. There was substantial variation in ability; 41% of nurses for normal delivery, 60% for post-partum hemorrhage and 45% for neonatal resuscitation provided less than half the correct actions. Ability declined with time after mentoring was completed.
Discussion: Mentoring improved the ability of ANMs to levels comparable with trained nurses. However, only some mentored nurses have the ability to conduct quality deliveries. Continuing education programs are critical to sustain quality gains.

Strengths and limitations of the study

- We were able to assess if the quality of obstetric care produced by auxiliary nurse-midwives (ANM), who typically serve as paramedical workers in rural communities in India, can be improved through on-site mentoring to levels comparable with trained staff nurses.
- This was a large scale on-site mentoring program that was implemented in around 340 (60%) primary care facilities and included both ANMs and staff nurses in the state of Bihar.
- Nurse ability measured using a unique tool that captures both knowledge and demonstrated skill.
- Observational study includes both intervention (mentored) and comparison (non-mentored) ANMs; however, no pre-intervention observations on nurse ability, but staff nurses serve as reference standard.
- ANM and staff nurse demonstrated ability to provide quality care can be different from what they do in actual practice.

For peer review only

INTRODUCTION

Access to a skilled birth attendant is a key strategy to reduce maternal and neonatal deaths in low and middle-income countries. India's high maternal mortality of 130 per 100,000 live births contributes 19% of global maternal deaths[1,2]. Institutional deliveries have been actively promoted by the government to increase access to skilled birth attendants; for example, since 2005 the government has offered cash incentives to pregnant women (and health workers at public facilities) if they deliver at a health facility. This has resulted in the institutional delivery rate doubling between 2005 and 2014 (from 39% to 79%)[3]. Notably, around 52% (54% in rural) of the deliveries nationwide occurred at a public sector facility; this highlights the prominent role of the public sector in providing women access to skilled birth attendants[3].

The remarkable increases in institutional deliveries in India have also raised concern about the quality of obstetric care. Studies have pointed out that despite substantial increases in institutional deliveries there has not been a concomitant reduction in maternal mortality, and its effect on neonatal mortality is unclear [4–10]. While the infrastructure and resources available at health facilities have been an ongoing challenge, more recently, attention has also focused on the performance of health care providers[11-14]. A recent study of nurses' management of obstetric complications at public facilities in India found that only 14% of nurses conducted initial assessments, 58% made a correct clinical diagnosis, and 20% provided first-line care[14]. A variety of factors constrain the ability of nurses to provide quality care – inadequate infrastructure, poor training, limited access to continuing education and professional support from experienced clinicians, lack of agency in the workplace, and entrenched detrimental clinical practices[6,15–18].

In health system contexts where trained clinicians are scarce, clinical functions are often shifted to health workers with less training and without appropriate support, which raises concerns about the quality of care [19]. In the state of Bihar where this study is situated, the lack of doctors and staff nurses at primary health centers (PHC) has resulted in lesser trained Auxiliary Nurse-midwives (ANM) providing obstetric care. Both staff nurses and ANMs are cadres within the public sector health system. Staff nurses have finished high school and have completed a diploma in General Nursing and Midwifery (GNM), a three year course in basic nursing [20]. GNM nurses are trained to conduct deliveries and manage complications. ANMs have completed high school and possess a two year diploma that trains them to be multi-purpose community health workers; their course work includes some training in managing deliveries [20]. Discussions the authors had with ANMs in Bihar indicate that their diploma programs include some course work but no practical training in managing deliveries. The curriculum of GNM and ANM programs is set by the Nursing Council of India [20]. ANMs were initially introduced into the health workforce in the 1950s to serve primarily as midwives at PHCs and sub-centers[21,22]. However, overtime their function changed from that of a midwife to a paramedical health worker who provides a range of services such as family planning, immunizations and some level of antenatal care[21,22]. In Bihar, because staff nurses are scarce at PHCs, ANMs have taken on the responsibility of managing deliveries.

Efforts to improve clinical skills of in-service nurses[1] have traditionally taken the form of short duration training sessions, often away from the health facility, which are not very effective for skill building. On-site clinical mentoring programs, which involve longer term engagement and relationship building between mentor and mentee, have shown to be effective in improving nurse knowledge and skills around essential obstetric and neonatal care in other countries, and

[1] We use the term nurses to include both ANMs and staff nurses.

For peer review only - http://bmjopen.bmj.com/site/about/guidelines.xhtml

more recently in India [23-27]. When mentors become part of the mentee's environment, mentoring functions such as didactic instruction, demonstrations, and support and encouragement, have the potential to improve mentee skills and self-efficacy to perform effectively and overcome challenges. On-site mentoring programs for nurses at PHCs in Bihar report increased nurse knowledge and management of normal and complicated deliveries, increased adherence to quality protocols during delivery, and increased confidence [26,27]. In other contexts mentoring was associated with a reduction in mortality for low birth weight babies[28].

This paper reports on a large-scale mentoring program to improve the quality of intrapartum care produced by ANMs (and staff nurses) at PHCs in the state of Bihar, India. Specifically, this study investigates if mentoring improved the ability of ANMs to manage normal and complicated deliveries; how mentored ANMs and staff nurses compared in ability, and the sustainability of the mentoring effect. Nurse ability was assessed in managing standardized cases of normal delivery, postpartum hemorrhage, and neonatal resuscitation. These cases were selected because mentees were taught to manage these conditions; hemorrhage is a leading cause of maternal mortality, and birth asphyxia a leading cause of neonatal mortality in India [29,30].

The state of Bihar (population 100 million) is among India's resource poor states. PHCs provide outpatient and occasionally inpatient care, maternal health services, family planning, public health services, and basic diagnostic and laboratory services. They cover between 20,000 to 30,000 people and are staffed with a doctor, nurses, and other supporting staff. PHCs are central to facility-based delivery of obstetric services because they are the first level in the public system which has clinicians trained to provide obstetric care such as doctors and nurses. In 2015-16, nearly 64% of all births were in health facilities and 36% at home; urban areas had 74% institutional births while rural areas had 63%[31]. Institutional births in public facilities have increased from just 4% of all institutional births in 2005-06 to almost 48% in 2015-16[31].

AMANAT nurse mentoring program in Bihar

CARE-India in collaboration with the Government of Bihar implemented the AMANAT on-site nurse mentoring program between March 2015 to January 2017. AMANAT aimed to improve the quality of basic emergency obstetric and newborn care (BEmONC) at primary health care facilities in the state of Bihar [32,33]. Because the nomenclature of primary care facilities is somewhat ambiguous in Bihar and change over time, we refer to AMANAT health facilities as PHCs in this study. The AMANAT program was preceded by a pilot from 2011 to 2013 in eight districts of Bihar [34]. The AMANAT program was implemented in a staggered manner over four phases; the program content was similar across phases [33]. In each phase 80 PHCs were purposively selected by CARE-India for mentoring. Several criteria determined selection of a PHC– availability of nurses or ANMs at the PHCs, the volume of deliveries, the infrastructural readiness of the PHC, the willingness of the PHC management to undertake mentoring, and the proximity of the PHC to other PHCs in the area such that mobile mentoring teams could easily rotate between them. In our analysis we include many of these selection criteria to construct a counterfactual set of health facilities. In total 400 out of 534 block-level (the smallest administrative unit within a district) PHCs in Bihar received the nurse mentoring intervention.

Prior to mentoring commencing at a PHC, ANMs and staff nurses affiliated with the particular PHC who were willing to participate in the mentoring were identified by the Medical Officer in-charge and requested to be present for the duration of mentoring. On average there were six to eight ANMs/staff nurses mentored in a PHC. In the AMANAT program, the same pair of

For peer review only - http://bmjopen.bmj.com/site/about/guidelines.xhtml

nurse-mentors rotated multiple times through four PHCs over a six to nine-month period, spending one consecutive week at a PHC when they visited (with an average of four PHCs for each nurse-mentor pair) [32]. The nurse-mentors were hired by CARE-Bihar, were recruited from across India, and had a BSc degree in nursing. The nurse-mentors were trained by CARE and provided with six days of training on simulation facilitation, team building, communication skills, and debriefing skills, followed by a four-day refresher training around three months into the mentoring period [35].

During their visit to the PHC, the nurse-mentors used structured learning sessions to provide didactic instruction covering a range of topics and bedside mentoring related to managing normal and complicated deliveries [32]. Nurses were trained on basic nursing procedures, infection prevention, basic obstetric and neonatal practices, management of complications such as post-partum hemorrhage, birth asphyxia, pre-eclampsia and others, documentation and reporting, team rapport and communication [32,33,35]. The typical sequence of mentoring sessions was - team building in the first week, followed by week long sessions on normal delivery and immediate newborn care, neonatal resuscitation and post-partum hemorrhage, and repeated mentoring on these conditions along with other obstetric and neonatal emergencies in the remaining weeks [33]. Tailored, structured simulations were used for normal deliveries and complications management through the PRONTOPack and other simulation kits[35-37]. These kits include MamaNatalie, a birth simulator worn by a demonstrator to resemble a pregnant woman and used for obstetric practices, and NeoNatalie, a neonate model used to demonstrate neonatal practices. Importantly, the nurse-mentors worked alongside mentees, observed them and provided instruction by demonstration through the co-management of cases. All mentoring and training materials were in the local Hindi language. Mentoring sessions were conducted in groups. ANMs and staff nurses were not mentored in separate groups. While a set of standard topics were covered during mentoring, certain aspects were emphasized to suit the particular learning needs of mentees and/or respond to clinical scenarios that emerged at the mentoring site.

METHODS

Study design

To assess the effect of mentoring on nurse ability we use a quasi-experimental post-test with matched comparison group design. PHCs exposed to AMANAT mentoring were matched on several criteria with non-mentored PHCs (see section Analytical Methods). The AMANAT program was implemented in four phases, such that, in each phase 80 PHCs were purposively selected. Because the nomenclature of primary care facilities is somewhat ambiguous and can include sub-district hospitals and other types of primary health care facilities, and these designations changes over time, we refer to AMANAT health facilities as PHCs in this study. Of the approximately 534 PHCs in Bihar, 80 PHCs were exposed to mentoring in a pilot phase (2011- 2013) and a further 320 were subsequently exposed during the AMANAT program (2015-2016). Therefore, around 134 PHCs were not exposed to mentoring. This study focuses on the 240 PHCs in phases 2, 3 and 4. These PHCs were first observed twice via repeat cross-sectional surveys – first, just after mentoring was completed and a second survey some months afterwards, depending on the phase. We excluded phase-1 PHCs because they had completed mentoring nearly two years prior to the survey date and we expected significant number of transfers of mentored nurses during this time.

Selection of PHCs: The 240 PHCs in phase 2,3 and 4 of the AMANAT program served as our sampling frame. For Phases 2 and 3, which began mentoring at approximately the same time, 40

For peer review only - http://bmjopen.bmj.com/site/about/guidelines.xhtml

PHCs from each phase were randomly sampled and a total of 79 out of these 80 selected PHCs were surveyed.[2] From Phase 4, 80 PHCS were selected and all were surveyed. Thus, a total of 159 mentored PHCs constitute the pool of intervention group PHCs.

134 PHCs in Bihar did not receive any mentoring either through the pilot or AMANAT program. Seven of these PHCs reported 15 or less deliveries per month in the state Health Management Information System (HMIS) and were excluded. The remaining 127 PHCs were mapped to assess geographical proximity to mentored facilities. From these PHCs, 80 PHCs that were closest to a mentored PHC – either from the same block or adjacent block as an intervention PHC - were purposively selected. These 80 PHCs form the pool of comparison group PHCs.

Selection of nurses: In the intervention PHCs, only ANMs and staff nurses who had completed mentoring were eligible to participate. For each sampled PHC the mentored staff nurses or ANMs were identified from mentoring rosters. From this list two were randomly selected and were requested to be present on the day the survey team arrived. From the 159 mentored PHCs a total of 314 staff nurses and ANMs were interviewed (out of a target of 318). From the comparison group of PHCs a convenience sample of two nurses were selected from among those present on the day the survey team arrived. From the 80 comparison PHCs 160 staff nurses and ANMs were interviewed (out of a target of 160).

Data collection and questionnaires: Between September 2016 and November 2017 a series of cross-sectional surveys of PHCs was conducted. PHCs and nurses in phases 2, 3 and 4 were first observed soon after mentoring was completed and the followed-up once after that. Phase 2 PHCs were visited 3 and 15 months after mentoring was completed, Phase 3 PHCs were visited 1 and 13 months after mentoring was completed and Phase 4 PHCs were visited just after and 6 months after mentoring was completed. Data was collected using tablets by trained enumerators who had a GNM or higher nursing degree. Enumerators were recruited from other parts of India and trained in Bihar for ten days to two weeks, depending on the particular batch of enumerators, on practices of managing normal and complicated deliveries, and administering the questionnaires.

Nurse ability was assessed using a structured questionnaire that captured both their knowledge and demonstrated skill in managing normal and complicated deliveries (see analytical methods). This questionnaire also included information on their background (age, number of months of AMANAT training completed, ANM or GNM nurse, regular or contract staff). Further, data from a cross-section survey of health facilities was carried out on the service delivery readiness of PHCs in Bihar and was used for matching intervention and comparison PHCs. In the PHCs surveyed information was collected on availability and condition of drugs, supplies, equipment, and building condition.

Ethical approval: Ethical clearance for the study was received from the Centre for Media Studies (New Delhi) Institutional Review Board vide approval number IRB00006230.

Patient and public involvement: We contacted four senior nurses in Bihar and elsewhere to guide development of the protocols and to train data collectors. The data collectors were also trained nurses. Feedback from nurses during pre-testing enabled designing study protocols so that they did not to place undue time burden on respondent nurses and were flexible to demands of their professional work.

[2] One facility in Phase 2 and 3 received the intervention in the initial pilot phase between Jan 2011 and December 2013 and was excluded.

For peer review only - http://bmjopen.bmj.com/site/about/guidelines.xhtml

Analytical methods

Matching health facilities: The intervention PHCs were selected on the basis of several criteria (see Background section). As such, there are likely to be systematic differences in these and other characteristics between intervention and comparison PHCs. Such observed and unobserved group differences can bias estimates of mentoring impact if they are related to the outcome of interest i.e. nurse ability. We adjust for this potential bias by matching intervention and comparison PHCs on several criteria. The health facility survey collected information on the service delivery readiness of PHCs in Bihar. Using the service delivery/readiness domains indicated in the WHO's Service Availability and Readiness Assessment (SARA)[38] as a guide, we identified the following six indicators for matching PHCs: location – connected to metaled road; basic amenities – power supply usually available or made available through a generator, hand washing station with running water available in labor room; drugs – oxytocin present; infrastructure – sanctioned bed strength; service utilization – average number of deliveries per year in 2016 (from HMIS).

We use Coarsened Exact Matching (CEM) to match PHCs in intervention and comparison groups[39]. After matching, 134 (84%) of the 159 AMANAT PHCs were matched with 56 (70%) of the 80 PHCs in the non-mentored group. From these matched PHCs, there were 265 nurses in the intervention group and 112 in the comparison group.

Measuring nurse ability: To measure nurse ability, we developed a tool that captures both their knowledge and demonstrated skill in managing normal and complicated deliveries. Our tool adapts elements of clinical vignettes with Objective Structured Clinical Examinations (OSCEs). Clinical vignettes have been used to measure health worker knowledge in treating specific disease conditions in the context of a simulated consultation[40-42]. Health care providers are presented with standardized cases specific to a disease condition and their knowledge in managing the specific condition is evaluated using checklists. OSCEs are commonly used across nursing and other allied health professions and involve learners rotating through structured, time-limited stations to demonstrate discrete skills[43,44]. OSCEs are used extensively in many countries to assess competencies in obstetric and newborn care, including in India[45-48]. Our tool combined clinical vignettes and OSCEs by evaluating knowledge (via verbal responses) and skills (via demonstration) in managing normal and complicated deliveries. Nurses and ANMs were evaluated in how they would manage three standardized cases - normal birth, neonatal resuscitation, and postpartum hemorrhage. These cases were selected because mentees were taught to manage these conditions; hemorrhage is a leading cause of maternal mortality, and birth asphyxia a leading cause of neonatal mortality in India [29,30]. Tool development was facilitated by CARE program developers, India-based obstetric experts, and international experts in BEmONC training. The same tool was used in all assessments. A panel of three nurses from the USA and India (Delhi and Bihar) assessed the face and content validity (by indicating if a particular item should be included or not) of the tool.

The three cases on which nurses and ANMs were evaluated – normal delivery, post-partum hemorrhage, and neonatal resuscitation – were structured to assess ability in history taking, examinations, case identification/diagnosis, and management. Two interviewers administered each case – interviewer #1 played the role of the pregnant woman and wore a Mama Natalie, and interviewer #2 observed the interaction using a standard checklist. Each case began with the respondent being presented with a situation by interviewer #2 – for example, the normal delivery case is introduced to the nurse/ANM as "*This is Tara [pointing to interviewer #1]. She is pregnant. She has come to see you at the clinic.*" The respondent was then asked what history questions she wanted to ask Tara, what examinations she would do, and the respondent demonstrated

For peer review only - http://bmjopen.bmj.com/site/about/guidelines.xhtml

them on the Mama Natalie. To each relevant history question or examination done, the respondent received a standard response. For instance, if the respondent asked "When did you have your last menstrual period (LMP)?" a standard response of "nine months" was offered or if the respondent demonstrated checking for fetal heart rate a response of "130" is given. No responses outside of the pre-structured conversation were given. Based on the results of the history and examinations, the nurse was asked to state the diagnosis/clinical decision (e.g. normal or complicated delivery), and then asked to use the Mama Natalie to demonstrate how she would manage the delivery. Each case comprised a set of items that corresponded to correct actions in the domains of history & examinations, case detection/diagnosis, and case management. All relevant correct actions performed was marked off on a checklist.

Data analysis: Each case comprises a set of items that correspond to correct actions necessary for managing the case. The unit of analysis is a necessary action (verbal or demonstration) provided by the respondent in the course of completing a case. Exploratory data analysis (e.g. histograms) was used to examine the distribution of provider responses across PHCs and nurses. Facilities with extreme values were identified and scrutinized. We then computed the proportion of relevant correct actions in history, examination, case detection/diagnosis, and management sections for each of the three cases. Next, using logistic regressions, for each case, we modelled the probability of a nurse providing a correct action as a function of mentoring status, nurse characteristics (GNM nurse (ref: ANM), age, permanent employee (ref: contractual), facility characteristics (connected to metaled road, regular power supply available or made available through a generator, hand washing station with running water available in labor room, oxytocin present; sanctioned bed strength, average number of deliveries per year in 2016, and item dummy variables. Interactions between nurse type and mentoring status were included. Regression standard errors were adjusted for clustering due to multiple observations on the same health facility. Goodness of fit test was performed on the final model by inspecting plots of predicted versus observed values. Residual plots were examined to identify outliers and influential observations. Regression results are presented in terms of marginal effects. To estimate changes in the mentoring effect over time we took advantage of the two cross-sectional surveys on the study PHCs - first, just after mentoring was completed and a second survey some months afterwards, depending on the phase. Locally weighted regressions (lowess) were estimated in which the binary outcome of a nurse providing a correct action was regressed on time (months) since the nurse completed mentoring. In reporting study results we followed STROBE guidelines for cross-sectional studies [49]. The statistical analysis was conducted using Stata 14 [50].

RESULTS

Table 1 shows the characteristics of the mentored and comparison PHCs and nurses in the matched and full sample. The full sample (see Total sample) includes the 239 mentored and comparison PHCs and 474 nurses before matching was carried out. After matching there were 134 mentored and 56 comparison PHCs, which yielded 256 mentored and 112 comparison nurses. The matched mentored and comparison PHCs are broadly similar in their characteristics indicating that matching was successful. However, mentored PHCs conducted more deliveries on average than the comparison ones. The PHCs and nurses in the full sample (i.e. Total sample in Table 1), are also broadly similar to the PHCs included in the matched sample in terms of PHC and nurse characteristics, though the matched PHCs have slightly higher values on all the PHC level indicators.

[Table 1 here]

For peer review only - http://bmjopen.bmj.com/site/about/guidelines.xhtml

There are several notable points about the characteristics of nurses. First, the overwhelming majority of nurses were ANMs; staff nurses were a minority. The mentored group had a higher proportion of ANMs relative to the comparison group. Secondly, the majority of nurses were regular staff (as opposed to contractual staff); their proportion is higher in the comparison group. Finally, nurses across groups are similar in average age. Nurse age is also correlated with service duration; nurses in the mentored and comparison groups had similar practice durations.

Nurses in the AMANAT program typically experienced six full months of mentoring. Overall 93% of nurses reported having at least six months of mentoring exposure (Table 1). Duration of mentoring ranged from two to nine months, with an average of seven months of mentoring. These results suggest that there was high adherence to the AMANAT program among those who participated.

ANM and Staff nurse ability

In all three cases mentored ANMs and staff nurses provided significantly more correct actions than the comparison group (Table 2). For example, mentored (comparison) ANMs provided 53% (36%) correct actions for the normal delivery case, a percentage point difference of 17 points; 49% (24%) for the post-partum hemorrhage case, a percentage point difference of 29 points, and 53% (25%) for the neonatal resuscitation case, a percentage point difference of 28 points. However, the overall proportion of correct actions among mentored ANMs and staff nurses does not exceed 60% in any case. Table 2 also shows the predicted probabilities from the regressions with respondent and PHC characteristics at their reference values. For example, for the normal delivery case mentored (comparison) ANMs provided 53% (36%) of the correct actions, while mentored staff nurses (comparison) provided 55% (42%) correct actions. Notably, the 95% confidence intervals between mentored and comparison nurses do not overlap - mentored ANMs and staff nurses had significantly higher percentage of correct actions compared to the comparison group.

[Table 2 here]

The ability of individual mentored nurses varied substantially. In the normal delivery case nurses provided between 20% to 90% of correct actions, in the post-partum hemorrhage case they provided between 11% to 89% of correct actions, and in the neonatal resuscitation case they provided between 6% and 95% of the correct actions (results not shown). Further, a substantial number of mentored nurses provided less than half the necessary correct actions required to manage these cases. For normal delivery 41%, post-partum hemorrhage 60% , and for neonatal resuscitation 45% of the nurses provided less than half the correct actions necessary for managing these cases.

Mentored ANMs and staff nurses demonstrated significantly more correct actions for history & examinations, and case management relative to those in the comparison group (Table 3). Mentored (comparison) ANMs provided 54% (41%) of the correct history and examination actions, 63% (34%) for post-partum hemorrhage and 55% (28%) for neonatal resuscitation. Similarly, mentored nurses performed better than the comparison group – mentored (comparison) ANMs provided 51% (28%) of correct case management actions for normal delivery, 37% (16%) for post-partum hemorrhage, and 51% (22%) for neonatal resuscitation. These patterns are also seen in the performance of staff nurses.

[Table 3 here]

For peer review only - http://bmjopen.bmj.com/site/about/guidelines.xhtml

The majority of ANMs and staff nurses were able to correctly identify cases (except for ANMs in the comparison group for neonatal resuscitation case) (Table 3). Expectedly, the percent of correct responses was highest for the normal delivery case since the pre-service training of both ANMs and staff nurses include the basics of managing normal delivery. However, the ability to correctly identify a case was higher among mentored nurses (Table 3). Mentored (comparison) ANMs correctly identified 96% (99%) of the normal delivery cases, 84% (51%) of the post-partum hemorrhage case, and 83% (50%) of the neonatal resuscitation case. Similarly, mentored staff nurses had higher case identification than the comparison group except for the normal delivery case. In general, staff nurses did much better than ANMs in correctly identifying a particular case.

Of interest is to see how well ANMs compare with staff nurses in their ability to manage normal and complicated deliveries. Comparison group (i.e. those without any mentoring) ANMs had significantly lower ability relative to staff nurses for all three cases. However, ANMs that experienced mentoring had similar levels of performance as staff nurses on all three cases. For example, for the normal delivery case mentored ANMs (staff nurses) provided 53% (55%) correct actions, 50% (50%) for post-partum hemorrhage, and 53% (57%) for neonatal resuscitation (Table 2). The overlapping confidence intervals between mentored ANMs and staff nurses indicates that there are no statistically significant differences between them.

Effect of mentoring

Mentored ANMs and staff nurses had significantly greater proportion, relative to the comparison group, of correct actions overall, and for the domains of history & examinations, case identification (except for normal delivery), and in case management (Table 2, 3). Figure 1 illustrates the adjusted mentoring effect (i.e. treatment effect on the treated) on ANMs and staff nurses. These are marginal effects from the regression models with other variables at their reference values. ANMs in the mentored group had 17.5% more correct actions for normal delivery relative to the comparison group, 25.9% more for post-partum hemorrhage, and 28.4% more for neonatal resuscitation. Staff nurses in the mentored group also show similar levels of improved performance. Notably, in all cases the mentoring effect is statistically significant i.e. the 95% confidence intervals do not overlap zero.

[Figure 1 here]

Both ANMs and staff nurses appear to have gained from mentoring, but ANMs have gained more. As seen in Figure 1, the mentoring effect for each case is greater for ANMs than staff nurses. Finally, because no differences in the proportion of correct actions are seen between mentored ANMs and staff nurses (Table 2), it appears that mentoring brought ANMs on par with staff nurses in the management of normal deliveries, post-partum hemorrhage, and neonatal resuscitation.

Of interest is to know if the mentoring effect on nurse ability changes with time. Figure 2 illustrates this by plotting estimates from local area regressions within the matched sample of PHCs; it shows the probability of nurses demonstrating a correct action at different points in time after mentoring was completed. Note that the same nurses are not observed at each point in time. In all three cases, nurse ability declined with time. In the normal delivery case, just after mentoring nurses were able to provide 53% of the correct actions, while nurses observed 43 months (the observation farthest from completion of mentoring) after mentoring were able to

For peer review only - http://bmjopen.bmj.com/site/about/guidelines.xhtml

provide 43% of correct actions, a ten-percentage point drop. Similar trends are seen in the case of neonatal resuscitation and postpartum hemorrhage. In the neonatal resuscitation case, the slight increase seen at the tail end appears to be an artifact of the data. For this case, nurses just after mentoring were able to provide 55% of the correct actions, which declined to 44% after 43 months post mentoring. For post-partum hemorrhage, just after mentoring nurses were able to provide 50% of correct actions, which fell to 37% 43 months post mentoring, a drop of 13 percentage points.

[Figure 2 here]

DISCUSSION

In India's public sector health system ANMs function as community based paramedical health workers and provide a range of services such as family planning, immunizations and antenatal care[21,22]. In human resource scarce states like Bihar, where there is a paucity of trained doctors and staff nurses, ANMs are the de-facto providers of obstetric care at many primary care facilities even though they are not formally trained for this role. In our study, in the 239 PHCs surveyed, staff nurses comprised only 16% of the sampled nurses. In a policy environment that aggressively promotes institutional deliveries the quality of care provided by ANMs is an important concern.

A principal finding from this study is that mentoring improved the average ability of ANMs and staff nurses at PHCs to manage normal and complicated deliveries. In all three cases – normal delivery, postpartum hemorrhage, and neonatal resuscitation – mentored ANMs and staff nurses had better average ability compared to non-mentored ones. This finding is consistent with evaluations of mentoring programs in other contexts and India [24-27]. Mentored ANMs (staff nurses) were able to provide 17% (13%) more correct actions for managing normal deliveries, 26% (14%) more for postpartum hemorrhage, and 28% (19%) more for neonatal resuscitation, compared to their non-mentored colleagues. The gains were greater for ANMs since they started from a lower level of ability relative to staff nurses. Among these three cases, for both ANMs and staff nurses the largest gain in ability was in neonatal resuscitation, followed by postpartum hemorrhage, and normal delivery. This is along expected lines since both ANMs and staff nurses are most familiar with the management of normal deliveries, so the incremental effect of mentoring would be relatively smaller. Further, the AMANAT mentoring program emphasized management of complications such as postpartum hemorrhage and neonatal resuscitation, which explains the gains observed in this area.

ANMs that had completed mentoring had the same average ability as staff nurses to manage normal and complicated deliveries. Further, un-mentored ANMs had significantly poorer ability than un-mentored staff nurses. This mentored ANM achievement is particularly salient because ANMs typically operate as paramedical health workers providing services such as family planning, immunizations and some level of antenatal care. It is unlikely that improvements in ANM ability would be achievable outside a mentoring context. The AMANAT program provided a combination of didactic teaching, supportive supervision, and professional support over a long period of time in the context of a mentor-mentee relationship. To the best of our knowledge there was no difference in the way staff nurses and ANMs were mentored, though the program was focused on ANMs given that they primarily manage deliveries at PHCs.

For peer review only

For peer review only - http://bmjopen.bmj.com/site/about/guidelines.xhtml

Mentoring improved case identification among ANMs. The vast majority of mentored ANMs were able to correctly identify a normal delivery case, 84% correctly identified a postpartum hemorrhage case, and 83% the neonatal resuscitation case. While non-mentored ANMs and staff nurses performed similarly as mentored ANMs in correctly identifying normal deliveries, they did quite poorly in identifying the other two cases. For example, among non-mentored ANMs, only half of them were able to correctly identify the postpartum hemorrhage or neonatal resuscitation case. The biggest improvement in correctly identifying a case was among ANMs for postpartum hemorrhage and neonatal resuscitation. The improvements observed in recognizing birth complications is important for identifying women for referral or complication management within the PHC.

Even as mentoring improved the average ability of nurses to provide quality care, there was substantial variation in nurse ability. Moreover, many mentored nurses were unable to provide a high level of correct actions. For example, 43% of the mentored nurses provided less than half the necessary actions in the normal delivery case. This suggests that participation in the mentoring program did not raise the ability of all nurses to some minimum standard of ability. There are several reasons for this, such as, the differential capacity of individual nurses to learn, the persistence of habit or features in the work environment that prevents nurses from imbibing new learnings, or low baseline knowledge levels that impedes advanced learning. An important implication is that exposure to mentoring (or any training program for that matter) does not automatically translate into improved ability to provide quality care for everyone. As such, it becomes important to assess how well mentored nurses provide quality care and only deploy those that meet some minimum quality standard.

There are several reasons why findings from this study might be generalizable to Bihar and to similar contexts. For one, the mentoring program was large-scale and covered most of the state. PHCs surveyed in the study covered the majority of the intervention PHCs and characteristics of the matched sample of PHCs and nurses aligns well with that of the larger pool.

It is important to be cautious about the extent to which quality of care can be improved by a single mentoring program. Even after six to nine months of mentoring through the AMANAT program there remained considerable scope for improving quality of care. Mentored ANMs and staff nurses were able to provide a little more than half the correct actions on all three cases. This is also reflected in the ability domains of history & examinations and case management on which nurses were evaluated. For example, mentored ANMs (nurses) were able to provide only 51% (54%) of correct actions for case management in the normal delivery case, 37% (38%) in the postpartum hemorrhage case, and 51% (56%) correct actions in the neonatal resuscitation case. Secondly, there will be variation in the ability of both ANMs and staff nurses post mentoring. Therefore, not all mentored ANMs and staff nurses will have the ability to properly manage normal or complicated deliveries. Moreover, the work environment can also affect nurse performance; the availability of adequate infrastructure, adequate staff, the management environment are all factors that can influence performance and modify the effect of mentoring.

Another reason for being cautious is that the mentoring effect on nurse ability appears to decay over time. In all three cases we find that nurse ability was highest in the period immediately after mentoring and this declined with the passage of time. The decay was least for the normal delivery case and most for the neonatal resuscitation case. This has also been observed in other nurse mentoring studies in India, which have documented declines in nurse practice and knowledge[26,51]. These patterns highlight the importance of continuing health worker education and make a strong case for mentoring or similar programs which are embedded within the health system to be offered on a frequent basis to health workers.

For peer review only

For peer review only - http://bmjopen.bmj.com/site/about/guidelines.xhtml

A limitation of this study is that we did not observe the ability of nurses before they started mentoring. As such, our estimates of the mentoring effect will be biased if the matched comparison group nurses do not have the same average ability as those in the mentored group before they were mentored. If at baseline comparison nurses had lower ability relative to non-mentored nurse, estimates of the mentoring effect will be upwardly biased. There are several reasons why this may not be the case. One source of bias could arise from differences in nurse characteristics between mentored and comparison groups that are related to their ability. Both mentored and comparison group ANMs (or staff nurses) had the same standard pre-service training and require the same qualifications to be recruited into government service. As such, it is unlikely that the extent or quality of nurse pre-service training differed between groups. Further, in our regression we control for difference in nurse and facility characteristics between groups. As such estimates of the mentoring effect are adjusted for differences in nurse characteristics such as age, years of service, and employee type between mentored and non-mentored nurses.

Nurse ability can vary by the characteristics of the environment where they work. For example, studies have noted that the ability of clinical care providers varies with the economic condition of the community i.e. better-off areas have better quality providers[52]. Similarly, nurses working in a facility located in economically better areas (e.g. close to urban areas) might have better ability. As such, differences in the facility location between the mentored and comparison groups can bias estimates of the mentoring effect on nurse ability. If mentored PHCs were located in more favorable areas then estimates of the mentoring effect will be upwardly biased. To address this we created the comparison group PHCs to be as similar as possible to the mentored PHCs in terms of location and physical environment. Comparison group PHCs were selected such that they were in close geographic proximity (from the same or adjacent administrative block) to the mentored PHCs. Further, we matched mentored and comparison PHCs on location, service readiness, and PHC use criteria.

Improved nurse ability need not translate into better practice in managing deliveries. Factors such as motivation, the work environment and support from colleagues, among others, mediate the pathway between ability to practice. However, studies that have examined the association between ability and practice report that these measures are correlated, though there is a typically a gap between what clinicians know and what they do in practice [53]. In general, more able clinicians also "do more" in practice, and ability measures the upper bound of what is possible in practice [53].

Mentoring programs can improve the quality of care of lesser trained health workers like ANMs to levels comparable to those of staff nurses who have higher levels of training. In health systems which struggle to recruit highly trained health workers to underserved areas, such mentoring programs offer a way of making task shifting strategies successful. However, other considerations like program costs and the ability to sustain such programs for long periods are also important for scale-up. To sustain the quality gains from mentoring it is important to expose health workers to regular continuing education programs.

For peer review only - http://bmjopen.bmj.com/site/about/guidelines.xhtml

ACKNOWLEDGEMENTS

The authors would like to acknowledge Priya Nanda (BMGF, Delhi), Yamini Atmavilas (BMGF Delhi), Dr. Sridhar Srikantiah (CARE-Bihar), Dr. Tanmay Mahapatra (CARE-Bihar), Tom Newton-Lewis (Oxford Policy Management, Delhi).

COMPETING INTERESTS

The authors declare they have no competing interests. AG and AD are employees of CARE-Bihar, the agency that designed and implemented the AMANAT program.

FUNDING

The study was funded by the Bill and Melinda Gates Foundation (https://www.gatesfoundation.org/) via grant number OPP1142884. The funders had no role in study design, data collection and analysis, decision to publish, or preparation of the manuscript.

AUTHORS CONTRIBUTIONS

The study was conceived and designed by KDR, NW, and SA. Study protocols were developed and by KDR, NW, SS, and KM. Field work was conducted by KDR, NW, SS, and KM. Data analysis was done by KDR, SS and SA. The paper was written primarily by KDR, SS and NW, with contributions from KM, AG, AD and SA.

DATA SHARING

The deidentified data used in this study can be obtained by writing to Krishna D. Rao (kdrao@jhu.edu) for purposes of replication.

For peer review only

REFERENCES

1. Sample Registration System. Special bulletin on maternal mortality in india 2014-16. Registrar General of India 2018. Available from: http://www.censusindia.gov.in/vital_statistics/SRS_Bulletins/MMR%20Bulletin-2014-16.pdf (accessed 3 Feb 2019)
2. World Health Organization. Trends in maternal mortality: 1990 to 2015: estimates by WHO, UNICEF, UNFPA, World Bank Group and the United Nations Population Division. Geneva; 2015. Available from: www.who.int/about/licensing/
3. International Institute for Population Sciences. National Family Health Survey-4 2015-16 – India fact sheet. Mumbai; Available from: http://rchiips.org/nfhs/pdf/NFHS4/India.pdf (accessed Apr 28 2018).
4. Randive B, Diwan V, De Costa A India's Conditional Cash Transfer Programme (the JSY) to Promote Institutional Birth: Is There an Association between Institutional Birth Proportion and Maternal Mortality? PLoS One 2013;8(6).
5. Sabde Y, Diwan V, Randive B, Chaturvedi S, Sidney K, Salazar M, et al. The availability of emergency obstetric care in the context of the JSY cash transfer programme in Madhya Pradesh, India. BMC Pregnancy Childbirth 2016;16.
6. Chaturvedi S, Randive B, Diwan V, De Costa A, Baradaran HR.Quality of Obstetric Referral Services in India's JSY Cash Transfer Programme for Institutional Births: A Study from Madhya Pradesh Province. PLoS One 2014;9(5).
7. Ameh C, Msuya S, Hofman J, Raven J, Mathai M, Van Den Broek N. Status of Emergency Obstetric Care in Six Developing Countries Five Years before the MDG Targets for Maternal and Newborn Health.PlosOne 2018. 2012;7(12)
8. Ng, M, Misra, A, Diwan, V, Agnani, M, Levin-Rector, A, & De Costa, A. An assessment of the impact of the JSY cash transfer program on maternal mortality reduction in Madhya Pradesh, India. *Global health action* 2014; *7*, 24939.
9. Powell-Jackson T, Mazumdar S, Mills A. Financial incentives in health: New evidence from India's Janani Suraksha Yojana. Journal of Health Economics 2015; 43: 154-169.
10. Lim SS, Dandona L, Hoisington JA, James SL, Hogan MC, Gakidou E. India's Janani Suraksha Yojana, a conditional cash transfer programme to increase births in health facilities: an impact evaluation. *Lancet 2010;* 375: 2009-2023
11. Mony PK, Krishnamurthy J, Thomas A, Sankar K, Ramesh BM, Moses S, et al. Availability and Distribution of Emergency Obstetric Care Services in Karnataka State, South India: Access and Equity Considerations. PLoS One 2013; 8(5).
12. Sharma G, Powell-Jackson T, Haldar K, Bradley J, Filippi V. Quality of routine essential care during childbirth: clinical observations of uncomplicated births in Uttar Pradesh, India. Bulletin of the World Health Organization 2017; 95(6):419–29.
13. Jayanna K, Mony P, Bm R, Thomas A, Gaikwad A, Hl M, et al. Assessment of facility readiness and provider preparedness for dealing with postpartum haemorrhage and pre-eclampsia/eclampsia in public and private health facilities of northern Karnataka, India: a cross-sectional study. BMC Pregnancy Childbirth 2014;14:304.
14. Chaturvedi S, Upadhyay S, De Costa A. Competence of birth attendants at providing emergency obstetric care under India's JSY conditional cash transfer program for institutional delivery: an assessment using case vignettes in Madhya Pradesh province. BMC Pregnancy Childbirth 2014; 14:174.
15. Evans CL, Maine D, McCloskey L, Feeley FG, Sanghvi H. Where there is no obstetrician - increasing capacity for emergency obstetric care in rural India: An evaluation of a pilot program to train general doctors. Int J Gynecol Obstet 2009; 107(3):277–82.
16. Iyengar SD, Iyengar K, Martines JC, Dashora K, Deora KK. Childbirth practices in rural

For peer review only - http://bmjopen.bmj.com/site/about/guidelines.xhtml

Rajasthan, India: Implications for neonatal health and survival. J Perinatol 2014; 28(SUPPL. 2):23–30.

17. Iyengar K, Jain M, Thomas S, Dashora K, Liu W, Saini P, et al. Adherence to evidence based care practices for childbirth before and after a quality improvement intervention in health facilities of Rajasthan, India. BMC Pregnancy Childbirth 2014;14(1):1–12.
18. Iyengar K, Iyengar SD. Emergency obstetric care and referral: experience of two midwife-led health centres in rural Rajasthan, India. Reprod Health Matters 2009; 17(33):9–20.
19. Rao KD, Sundararaman T, Bhatnagar A, Gupta G, Kokho P, Jain K. Which doctor for primary health care? Quality of care and non-physician clinicians in India. Soc Sci Med 2013;84:30–4.
20. Indian Nursing Council. Retreived from: http://www.indiannursingcouncil.org/nursing-programs.asp?show=elig-crit.
21. Mavalankar D, Vora KS. The Changing Role of Auxiliary Nurse Midwife (ANM) in India : Implications for Maternal and Child Health. 2008; Indian Institute of Management Ahmedabad, Research and Publication Department, IIMA Working Papers.
22. Malik G. Role of auxiliary nurse midwives in National Rural Health Mission. Nurs J India 2009; 100(4): 88-91.
23. Anatole M, Magge H, Redditt V, Karamaga A, Niyonzima S, Drobac P, et al. Nurse mentorship to improve the quality of health care delivery in rural Rwanda. Nurs Outlook 2013;61(3):137–44.
24. Fischer EA, Jayana K, Cunningham T, Washington M, Mony P, Bradley J, et al. Nurse Mentors to Advance Quality Improvement in Primary Health Centers: Lessons From a Pilot Program in Northern Karnataka, India. Glob Health Sci Pract. 2015 Dec 17;3(4):660-75.
25. Jayanna K, Bradley J, Mony P, Cunningham T, Washington M, Bhat S, et al. Effectiveness of Onsite Nurse Mentoring in Improving Quality of Institutional Births in the Primary Health Centres of High Priority Districts of Karnataka, South India: A Cluster Randomized Trial. PlosOne 2016; 11(9)
26. Das A, Nawal D, Singh MK, Karthick M, Pahwa P, Shah MB, et al. Evaluation of the mobile nurse training (MNT) intervention - a step towards improvement in intrapartum practices in Bihar, India. BMC Pregnancy Childbirth 2017;17(1):1–11.
27. Das A, Nawal D, Singh MK, Karthick M, Pahwa P, Shah MB, et al. Impact of a Nursing Skill-Improvement Intervention on Newborn-Specific Delivery Practices: An Experience from Bihar, India. Birth 2016;43(4):328–35.
28. Bellad RM, Bang A, Carlo WA, McClure EM, Meleth S, Goco N, et al. A pre-post study of a multi-country scale up of resuscitation training of facility birth attendants: Does Helping Babies Breathe training save lives? BMC Pregnancy Childbirth. 2016;16(1):1–10.
29. Montgomery AL, Ram U, Kumar R, Jha P, for The Million Death Study Collaborators. Maternal Mortality in India: Causes and Healthcare Service Use Based on a Nationally Representative Survey. PLoS ONE 2014; 9(1)
30. Million Death Study Collaborators, Bassani, DG, Kumar R, Awasthi S, Morris SK, Paul VK, Shet A, Ram U, Gaffey MF, Black RE, et al. Causes of neonatal and child mortality in India: a nationally representative mortality survey. *Lancet 2010*; 376(9755):1853-60.
31. International Institute for Population Sciences. National Family Health Survey-4. State Fact Sheet Bihar 2015-16. 2015; Available from: http://rchiips.org/nfhs/factsheet_NFHS-4.shtml
32. CARE India. A model for mentorship [Internet]. 2018. Available from http://bihar.care.org/wp-content/uploads/2018/08/Bihar_4_REV_SCREEN.pdf
33. Vail B, Spindler H, Morgan MC, Cohen SR, Christmas A, Sah P, Shah MB, Das A, et al. Care of the mother-infant dyad: a novel approach to conducting and evaluating neonatal

For peer review only - http://bmjopen.bmj.com/site/about/guidelines.xhtml

resuscitation simulation training in Bihar, India. BMC pregnancy and childbirth 2017; *17*(1), 252.

34. CARE India. Integrated Family Health Initiative: Catalysing change for healthy communities [Internet]. 2012. Available from: http://www.care.org/sites/default/files/documents/MH-2013-BIHAR_IFHI_Program Summary.pdf
35. Vail B, Morgan MC, Spindler H, Christmas A, Cohen SR, & Walker DM. The power of practice: simulation training improving the quality of neonatal resuscitation skills in Bihar, India. BMC pediatrics 2018; 18(1), 291.
36. Morgan MC, Dyer J, Abril A, Christmas A, Mahapatra T, Das A, and Walder DM. Barriers and facilitators to the provision of optimal obstetric and neonatal emergency care and to the implementation of simulation-enhanced mentorship in primary care facilities in Bihar, India: a qualitative study. BMC Pregnancy and Childbirth 2018; 18:42
37. Raney, JH, Morgan MC, Christmas A, et al. Simulation-enhanced nurse mentoring to improve preeclampsia and eclampsia care: an education intervention study in Bihar, India. BMC Pregnancy Childbirth 2019; 19: 41.
38. World Health Organization. Service Availability and Readiness Assessment (SARA) An annual monitoring system for service delivery Reference Manual.
39. Iacus SM, King G, Porro G. Causal inference without balance checking: Coarsened exact matching. Polit Anal. 2012;20(1):1–24.
40. Das J, Hammer J. Which doctor? Combining vignettes and item response to measure clinical competence. J Dev Econ. 2005;78(2):348–83.
41. Leonard KL, Masatu MC. The use of direct clinician observation and vignettes for health services quality evaluation in developing countries. Soc Sci Med 2005;61(9):1944–51. A
42. Peabody JW. Measuring the Quality of Physician Practice by Using Clinical Vignettes: A Prospective Validation Study. Ann Intern Med 2004; 141(10):771.
43. Rushforth HE. Objective structured clinical examination (OSCE): Review of literature and implications for nursing education. Nurse Educ Today 2007; 27(5):481–90.
44. Patrício MF, Julião M, Fareleira F, Carneiro AV. Is the OSCE a feasible tool to assess competencies in undergraduate medical education? Med Teach 2013;35(6):503–14.
45. Aimable M, Birgitta E, Cyprien B, Alphonse R, Lars-Åke P. Evaluating Helping Babies Breathe: training for healthcare workers at hospitals in Rwanda. Acta Paediatr 2012;102(1):e34–8.
46. Thukral A, Lockyer J, Bucher SL, Berkelhamer S, Bose C, Deorari A, et al. Evaluation of an educational program for essential newborn care in resource-limited settings: Essential Care for Every Baby. BMC Pediatr 2015;15(1)
47. Goudar SS, Dhaded SM, McClure EM, Derman RJ, Patil VD, Mahantshetti NS, et al. ENC training reduces perinatal mortality in Karnataka, India. J Matern Neonatal Med 2012;25(6):568–74.
48. Varghese B, Krishnamurthy J, Correia B, Panigrahi R, Washington M, Ponnuswamy V, et al. Limited Effectiveness of a Skills and Drills Intervention to Improve Emergency Obstetric and Newborn Care in Karnataka, India: A Proof-of-Concept Study. Glob Heal Sci Pract 2016;4(4).
49. von Elm E1, Altman DG, Egger M, Pocock SJ, Gøtzsche PC, Vandenbroucke JP; STROBE Initiative. The Strengthening the Reporting of Observational Studies in Epidemiology (STROBE) statement: guidelines for reporting observational studies. Bull World Health Organ 2007;85(11):867-72.
50. StataCorp. 2015. *Stata Statistical Software: Release 14*. College Station, TX: StataCorp LP.
51. Bang A, Patel A, Bellad R, Gisore P, Goudar SS, Esamai F, et al. Helping Babies Breathe (HBB) training: What happens to knowledge and skills over time? BMC Pregnancy Childbirth 2016; 16(1):364.

For peer review only - http://bmjopen.bmj.com/site/about/guidelines.xhtml

52. Das J, Mohpal A. Socioeconomic Status And Quality Of Care In Rural India: New Evidence From Provider And Household Surveys. Health Aff 2016;35(10):1764–73.

53. Leonard K, Masatu M. Professionalism and the know-do gap: exploring intrinsic motivation among health workers in Tanzania. Health Econ 2010;19(12):1461–77.

For peer review only

For peer review only - http://bmjopen.bmj.com/site/about/guidelines.xhtml

FIGURE LEGENDS

Figure 1: Effect of mentoring on ability of ANMs and Staff nurses

Figure 2: Trends in nurse ability after completion of mentoring (ANM and Staff Nurse combined, lowess estimates)

For peer review only

Table 1: Characteristics of matched and unmatched sample

Indicator	Matched PHCs		Total sample*
	Mentored	Comparison	
Primary Health Centers			
N (PHC)	134	56	239
Connected to metaled road (%)	92	87	89
Electricity always available (%)	90	89	86
Labor room has functional handwashing (%)	100	100	93
Oxytocin available (%)	83	75	79
Average number of beds	10.9 (10.95)	10.2 (14.44)	15.3 (30.19)
Average number of deliveries in past year	193.4 (93.91)	133.8 (93.39)	170.6 (101.11)
Nurses			
N(staff nurses & ANMs)	265	112	474
Completed full course of mentoring (%)	93	-	-
ANMs (%)	89	87	84
Regular (non-contractual) staff (%)	63	71	67
Age (years)	41.1 (8.75)	43.2 (9.70)	41.8 (9.22)

Note: (1) *The 'Total sample' is the pool of mentored (159) and non-mentored (80) PHCs from where the matched sample of mentored (134) and non-mentored (56) PHCs was drawn. Consequently, because some mentored and comparison PHCs were unmatched, the Total Sample does not equal the sum of matched mentored and non-mentored PHCs. (2) Figures in parenthesis are SD.

For peer review only - http://bmjopen.bmj.com/site/about/guidelines.xhtml

Table 2: Overall ability of staff nurse and auxiliary nurse-midwife (ANM) to manage normal and complicated deliveries

	Normal Delivery		Post-Partum Hemorrhage		Neonatal Resuscitation	
	M	C	M	C	M	C
Auxiliary Nurse Midwife (ANM)						
N (ANMs)	237	98	237	98	237	98
n (items)	11850	4493	4266	1617	3792	1438
% of correct actions (unadjusted)	53	36	49	24	53	25
Predicted probability of correct action	0.53	0.36	0.50	0.25	0.53	0.24
95% CI	[0.51,0.55]	[0.33,0.38]	[0.48,0.53]	[0.22,0.28]	[0.49,0.56]	[0.19,0.30]
Staff nurse						
N (staff nurse)	28	14	28	14	28	14
n (items)	1400	1108	504	399	448	354
% of correct actions (unadjusted)	55	41	48	33	58	36
Predicted probability of correct action	0.55	0.42	0.50	0.37	0.57	0.38
95% CI	[0.51,0.59]	[0.37,0.48]	[0.44,0.56]	[0.31,0.42]	[0.49,0.66]	[0.31,0.46]

Note: Predicted probabilities are the probability that a staff nurse/ANM provide a correct action, with all other variables in the regression at their reference value. Multiplying these figures by 100 gives the percentage of correct actions provided by the staff nurse/ANM.
M: Mentored; C: Comparison

For peer review only

Table 3: Correct actions provided by auxiliary nurse-midwives (ANM) and staff nurses by case domains.

	**Normal Delivery**		**Post-Partum Hemorrhage**		**Neonatal Resuscitation**	
	M	C	M	C	M	C
Auxiliary Nurse Midwife (ANM), % (items)						
N(ANMs)	237	98	237	98	237	98
Correct history & examination	54* (5,925)	41 (2,246)	63* (1,422)	34 (539)	55* (474)	28 (180)
Correct case identification	96 (237)	99 (90)	84* (237)	51 (90)	83* (237)	50 (90)
Correct case management actions	51* (5,688)	28 (2,156)	37* (2,607)	16 (988)	51* (3,081)	22 (1,168)
Staff nurses, % (items)						
N (staff nurses)	28	14	28	14	28	14
Correct history & examination	55* (700)	41 (554)	63* (168)	46 (133)	48 (56)	28 (44)
Correct case identification	96 (28)	100 (22)	89 (28)	76 (22)	100* (28)	75 (22)
Correct case management actions	54* (672)	38 (531)	38* (308)	22 (244)	56* (364)	34 (288)

*Chi-sq test p-value<0.05; M: Mentored; C: Comparison

For peer review only

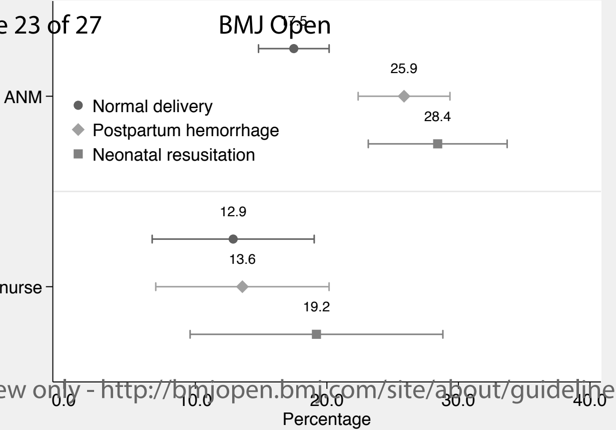

Note: Figures show the percentage point difference (and 95% CI) in quality actions undertaken between the mentored and non-mentored group adjusted for other variables

er review only - http://bmjopen.bmj.com/site/about/guidelines

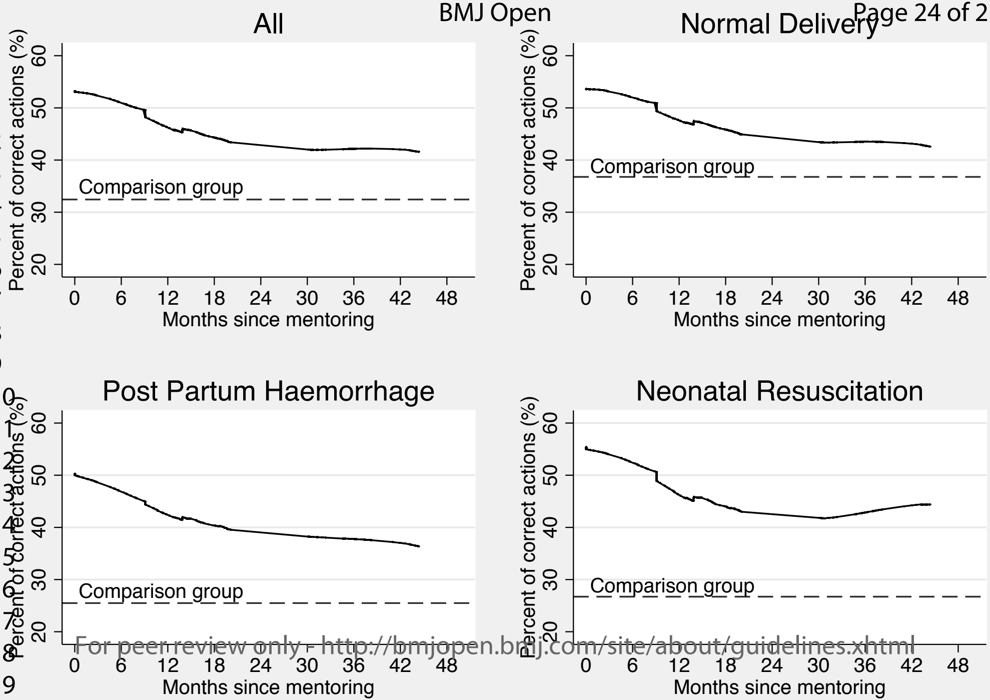

For peer review only - http://bmjopen.bmj.com/site/about/guidelines.xhtml

STROBE 2007 (v4) Statement—Checklist of items that should be included in reports of *cross-sectional studies*

Section/Topic	Item #	Recommendation	Reported on page #/section
**Title and abstract**	1	(*a*) Indicate the study's design with a commonly used term in the title or the abstract	abstract
		(*b*) Provide in the abstract an informative and balanced summary of what was done and what was found	abstract
**Introduction**			
Background/rationale	2	Explain the scientific background and rationale for the investigation being reported	Background, page 1
Objectives	3	State specific objectives, including any prespecified hypotheses	Background, page 2
**Methods**			
Study design	4	Present key elements of study design early in the paper	Methods, page 3
Setting	5	Describe the setting, locations, and relevant dates, including periods of recruitment, exposure, follow-up, and data collection	Background, Page 2; Methods, Page 3&4
Participants	6	(*a*) Give the eligibility criteria, and the sources and methods of selection of participants	Methods, Page 3&4
Variables	7	Clearly define all outcomes, exposures, predictors, potential confounders, and effect modifiers. Give diagnostic criteria, if applicable	Outcome: Methods, Page 4; Exposure: Background, page 1&2
Data sources/ measurement	8*	For each variable of interest, give sources of data and details of methods of assessment (measurement). Describe comparability of assessment methods if there is more than one group	Outcome: Methods, Page 4/5; Other variables Page 4.
Bias	9	Describe any efforts to address potential sources of bias	*Page 4*
Study size	10	Explain how the study size was arrived at	Page 4
Quantitative variables	11	Explain how quantitative variables were handled in the analyses. If applicable, describe which groupings were chosen and why	Page 5
Statistical methods	12	(*a*) Describe all statistical methods, including those used to control for confounding	Page 4 & 5
		(*b*) Describe any methods used to examine subgroups and interactions	Page 5
		(*c*) Explain how missing data were addressed	No missing data

For peer review only

		(*d*) If applicable, describe analytical methods taking account of sampling strategy	N/a
		(*e*) Describe any sensitivity analyses	N/a
**Results**			
Participants	13*	(a) Report numbers of individuals at each stage of study—eg numbers potentially eligible, examined for eligibility, confirmed eligible, included in the study, completing follow-up, and analysed	Page 6
		(b) Give reasons for non-participation at each stage	N/a
		(c) Consider use of a flow diagram	N/a
Descriptive data	14*	(a) Give characteristics of study participants (eg demographic, clinical, social) and information on exposures and potential confounders	Page 6, Table 1
		(b) Indicate number of participants with missing data for each variable of interest	None
Outcome data	15*	Report numbers of outcome events or summary measures	Page 6, Table 2,3
Main results	16	(*a*) Give unadjusted estimates and, if applicable, confounder-adjusted estimates and their precision (eg, 95% confidence interval). Make clear which confounders were adjusted for and why they were included	Table 2, 3; Page 5,6, Figure 1
		(*b*) Report category boundaries when continuous variables were categorized	N/a
		(*c*) If relevant, consider translating estimates of relative risk into absolute risk for a meaningful time period	N/a
Other analyses	17	Report other analyses done—eg analyses of subgroups and interactions, and sensitivity analyses	Interactions Page 6
**Discussion**			
Key results	18	Summarise key results with reference to study objectives	Page 8,9
Limitations	19	Discuss limitations of the study, taking into account sources of potential bias or imprecision. Discuss both direction and magnitude of any potential bias	Page 9
Interpretation	20	Give a cautious overall interpretation of results considering objectives, limitations, multiplicity of analyses, results from similar studies, and other relevant evidence	Page 9 and 10
Generalisability	21	Discuss the generalisability (external validity) of the study results	Page 9
**Other information**			
Funding	22	Give the source of funding and the role of the funders for the present study and, if applicable, for the original study on which the present article is based	Page 11

*Give information separately for cases and controls in case-control studies and, if applicable, for exposed and unexposed groups in cohort and cross-sectional studies.

For peer review only

Note: An Explanation and Elaboration article discusses each checklist item and gives methodological background and published examples of transparent reporting. The STROBE checklist is best used in conjunction with this article (freely available on the Web sites of PLoS Medicine at http://www.plosmedicine.org/, Annals of Internal Medicine at http://www.annals.org/, and Epidemiology at http://www.epidem.com/). Information on the STROBE Initiative is available at www.strobe-statement.org.

For peer review only
